# A Chromosome-Level Genome Assembly of the Anglerfish *Lophius litulon*

**DOI:** 10.3389/fgene.2020.581161

**Published:** 2020-11-27

**Authors:** Meiqi Lv, Yaolei Zhang, Kaiqiang Liu, Chang Li, Jiahao Wang, Guangyi Fan, Xin Liu, Huanming Yang, Changlin Liu, Shahid Mahboob, Junnian Liu, Changwei Shao

**Affiliations:** ^1^School of Future Technology, University of Chinese Academy of Sciences, Beijing, China; ^2^BGI-Qingdao, BGI-Shenzhen, Qingdao, China; ^3^State Key Laboratory of Agricultural Genomics, BGI-Shenzhen, Shenzhen, China; ^4^Department of Biotechnology and Biomedicine, Technical University of Denmark, Lyngby, Denmark; ^5^Key Lab of Sustainable Development of Marine Fisheries, Ministry of Agriculture, Yellow Sea Fisheries Research Institute, Chinese Academy of Fishery Sciences, Qingdao, China; ^6^Laboratory for Marine Fisheries Science and Food Production Processes, Qingdao National Laboratory for Marine Science and Technology, Qingdao, China; ^7^BGI-Shenzhen, Shenzhen, China; ^8^Department of Zoology, College of Sciences, King Saud University, Riyadh, Saudi Arabia; ^9^Qingdao-Europe Advanced Institute for Life Sciences, BGI-Shenzhen, Qingdao, China

**Keywords:** anglerfish, *Lophius litulon*, Lophiiformes, chromosomal evolution, metabolism

## Abstract

Anglerfishes are a highly diverse group of species with unique characteristics. Here, we report the first chromosome-level genome of a species in the order Lophiiformes, the yellow goosefish (*Lophius litulon*), obtained by whole genome shotgun sequencing and high-throughput chromatin conformation capture. Approximately 97.20% of the assembly spanning 709.23 Mb could be anchored to 23 chromosomes with a contig N50 of 164.91 kb. The BUSCO value was 95.4%, suggesting that the quality of the assembly was high. A comparative gene family analysis identified expanded and contracted gene families, and these may be associated with adaptation to the benthic environment and the lack of scales in the species. A majority of positively selected genes were related to metabolic processes, suggesting that digestive and metabolic system evolution expanded the diversity of yellow goosefish prey. Our study provides a valuable genetic resource for understanding the mechanisms underlying the unique features of the yellow goosefish and for investigating anglerfish evolution.

## Introduction

Lophiiformes (commonly known as anglerfishes) is a teleost order containing 68 genera and more than ∼300 species found in benthic, shallow-water, and deep sea habitats (Miya et al., 2010). Yellow goosefish (*Lophius litulon*) belonging to Lophiiformes is mainly distributed in seas of Korea, Japan, and China. It is an important commercial fish and is widely consumed. Various characteristics of Lophiiformes, including the functional morphology (Farina and Bemis, 2016), geographic distribution, population structure, migration, feeding ecology, and reproduction of species (Farina et al., 2008), have been investigated. Additionally, phylogenetic relationships in Lophiiformes have been studied by using mitochondrial genomes of 39 species from 33 genera (Miya et al., 2010). However, high-quality whole genome sequences for species in this order are not available, limiting our understanding of the molecular basis of the unique characteristics of this group, such as the lack of scales on the body surface, diverse feeding habits, and large liver.

In this study, we sampled a female yellow goosefish from the Yellow Sea, China, and generated a chromosome-level genome assembly. To the best of our knowledge, this is the first chromosome-level genome for Lophiiformes. We performed a comparative genomic analysis, including analyses of repeat contents, gene families, and positive selection, to understand the genetic basis of the unique characteristics of the species.

## Materials and Methods

### DNA and RNA Sequencing

**FIGURE 1:**
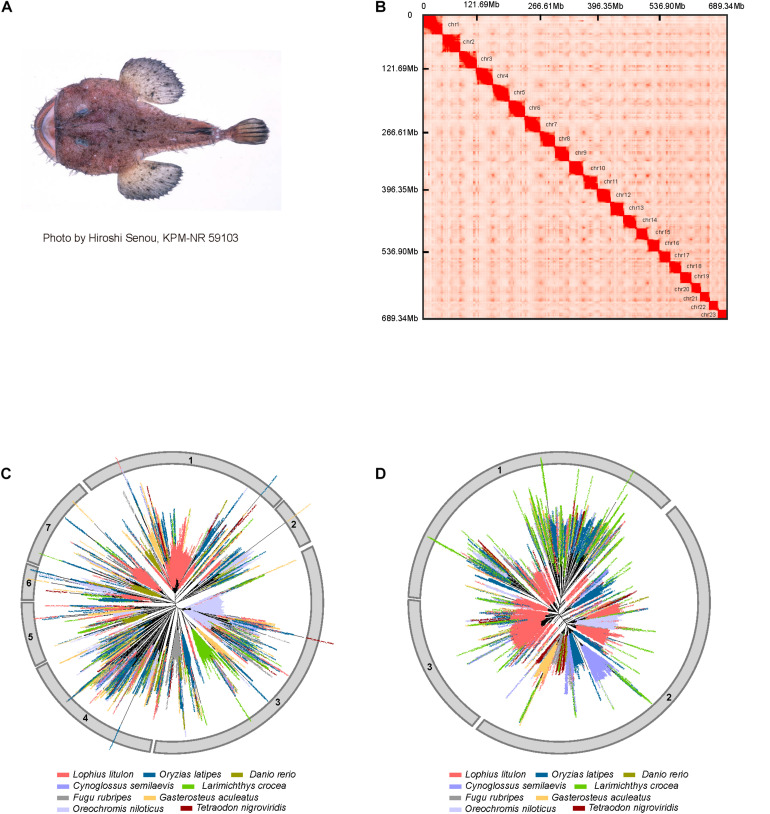
Genome assembly and expansion of LINEs in yellow goosefish. **(A)** Example of a yellow goosefish (*Lophius litulon*). **(B)** 23 chromosomes contact maps of the yellow goosefish genome. The blocks represent the contact between locations, and the color represents the intensity of each contact. **(C)** Phylogenetic tree of LINE/L2. **(D)** Phylogenetic tree of LINE/RTE-BovB.

The yellow goosefish used in this study was caught in the Yellow Sea near Qingdao, China, by the Yellow Sea Fisheries Research Institute. Genomic DNA was extracted from muscle tissues of a female yellow goosefish (Figure 1A) and processed according to a previously described protocol (Shao et al., 2018). The Qubit 3.0 fluorometer and gel electrophoresis were used to evaluate the DNA quantity and integrity, respectively. Four whole genome sequencing libraries, including one paired-end library with an insert size of 350 bp and three mate-pair libraries with insert sizes of 2, 5, and 10 kb, were constructed by standard protocols (Shao et al., 2018) and sequenced using the BGISEQ-500 platform. SOAPnuke (v1.5.6) (Chen Y. et al., 2018) was used to filter raw data with the parameters “filter -d -n 0.1 -l 10 -q 0.5 -Q 2” to obtain high-quality data. A Hi-C library (Lieberman-Aiden et al., 2009) was also constructed using a blood sample according to a previously described protocol (Shao et al., 2018) and sequenced using the BGISEQ-500 platform, yielding paired-end reads with lengths of 100 bp. Quality control of Hi-C data was performed using the HIC-Pro pipeline (v2.11.1) (Servant et al., 2015) with default parameters.

RNA was extracted from 11 tissues (liver, kidney, spleen, spina dorsalis, heart, ovary, muscle, eye, skin, brain, and blood) of four female yellow goosefishes using TRIzol Reagent (Invitrogen, Waltham, MA, United States). Paired-end libraries with insert sizes of 350 bp were sequenced on the BGISEQ-500 platform, and the sequencing data were filtered using SOAPnuke (v1.5.6) with the parameters “filter -l 15 -q 0.2 -n 0.05 -Q 2.”

### Genome Assembly

To estimate the genome size of the yellow goosefish, a *k*-mer analysis (*k* = 17) (Li et al., 2010) was conducted. The genome was assembled using clean sequence data from different insert size libraries (350 bp, 2 kb, 5 kb, and 10 kb) using ABySS (v2.0.2) (Nielsen et al., 2009) with the parameters “abyss-pe k = 63.” To further improve the quality of the assembly, krskgf (v. 1.19) (Zhang et al., 2014) was used setting kmer = 41 and Gapcloser (v1.10) (Luo et al., 2012) was used with default parameters to fill gaps. Allelic scaffolds were removed using the Redundans pipeline (Pryszcz and Gabaldon, 2016) with default parameters. Finally, valid reads were obtained from raw data using the HiC-Pro pipeline with default parameters to generate the chromosomal-level assembly. Juicer (v. 1.5) (Durand et al., 2016) was used to analyze Hi-C datasets and the 3D-DNA pipeline (v. 170123) (Dudchenko et al., 2017) was used to anchor the yellow goosefish genome assembly to chromosomes with the parameters “-m haploid -s 4.”

For topologically associated domains calling, pair-end reads were aligned to genome by Bowtie2, with the parameters “-set –local –reorder.” HicBuildMatrix tool (v2.2.3) (Wolff et al., 2018) was used to build the matrix of read counts over the bins in the genome with bin size of 5,000 bp, considering the sites around the given restriction site. Then eight 5 kb bins were merged into 40 kb bins matrix by hicMergeMatrixBins (v2.2.3). The Hi-C matrix was corrected by hicCorrectMatrix (v2.2.3) after removing bins with lower number of reads. The boundaries were identified and final TADs were detected by hicFindTADs.

### Genome Annotation

Two methods were combined for repeat content annotation. Homolog-based searches against the Repbase (v21.01) database (Jurka et al., 2005) using RepeatMasker (v4.0.6) and RepeatProteinMask (v4.0.6) with the parameters “-nolow -no_is -norna -engine ncbi” and “-engine ncbi -noLowSimple -pvalue 0.0001” were first performed. Then, *de novo* prediction was performed using RepeatModeler (v1.0.8) and LTR-FINDER (v1.0.6) (Xu and Wang, 2007). Tandem Repeats Finder (v4.07) (Benson, 1999) (with the parameters “-Match 2 -Mismatch 7 -Delta 7 -PM 80 -PI 10 -Minscore 50 -MaxPeriod 2000”) was used to detect tandem repeats. Additionally, repeats in another eight representative fish genomes were evaluated, including the tongue sole (*Cynoglossus semilaevis*), zebrafish (*Danio rerio*), stickleback (*Gasterosteus aculeatus*), large yellow croaker (*Larimichthys crocea*), medaka (*Oryzias latipes*), tilapia (*Oreochromis niloticus*), spotted green pufferfish (*Tetraodon nigroviridis*), and fugu (*Takifugu rubripes*), using the same pipelines. The genome sequences of these eight species were downloaded from the National Center for Biotechnology Information (NCBI) database and ENSEMBL (release-84). To study Bovine-B (BovB) and L2 retrotransposons, dynamic families of long interspersed nuclear elements (LINEs) in eukaryotes (Ichiyanagi and Okada, 2008; Ivancevic et al., 2018), 1% of BovB repeats, and 1% of L2 repeats were randomly extracted from our annotation results, respectively. These were used to generate multiple sequence alignments respectively using MAFFT (v7.245) (Katoh and Standley, 2013). Finally, the phylogenetic trees of BovB and L2 elements were constructed respectively using FastTree (v2.1.10) by maximum likelihood method (Price et al., 2010).

For gene annotation, gene structures were predicted using three methods. For *de novo* prediction, Augustus (v3.1) (Stanke et al., 2006) and GENSCAN (v2.1) (Burge and Karlin, 1997) were applied to the repeat-masked genome. Protein sequences of six species were downloaded from the NCBI database for homolog-based prediction. The six species were the tongue sole (*Cynoglossus semilaevis*), zebrafish (*Danio rerio*), large yellow croaker (*Larimichthys crocea*), tilapia (*Oreochromis niloticus*), medaka (*Oryzias latipes*), and fugu (*Takifugu rubripes*). BLAT (v35.1) (Kent, 2002) was used to map the protein sequences to the yellow goosefish genome and then GeneWise (v2.4.1) (Birney et al., 2004) was used to obtain gene models. For transcriptome annotation, the filtered transcriptome reads were assembled using Trinity (v2.0.6) (Grabherr et al., 2011) and the assembly was mapped to the yellow goosefish genome using BLAT (v35.1) and PASA to obtain coding sequence models. Finally, all results were integrated to obtain a non-redundant gene set using GLEAN (Elsik et al., 2007). The final gene sets were assessed using BUSCO with the actinopterygii database. For gene function annotation, protein sequences were aligned to several databases, including Swiss-Prot (Bairoch and Apweiler, 2000), TrEMBL (Bairoch and Apweiler, 2000), and Kyoto Encyclopedia of Genes and Genomes (KEGG v84.0) (Kanehisa and Goto, 2000), using BLASTP (v2.6.0+) (Altschul et al., 1990) with an *E*-value threshold of 10^–5^. InterProScan (v5.16-55.0) (Zdobnov and Apweiler, 2001) was used to determine function-specific motifs and domains in protein databases, including Pfam, SMART, PANTHER, PRINTS, PROSITE profiles, ProDom, and ProSitePatterns. Gene Ontology (GO) annotation results were extracted from the InterProScan results.

### Synteny Analysis With Fugu

A whole genome alignment between yellow goosefish and fugu (*Takifugu rubripes*) genomes was generated using LASTZ (v1.1) (Harris, 2007) with the parameter settings “T = 2 C = 2 H = 2000 Y = 3400 L = 6000 K = 2200.” After filtering the aligned blocks shorter than 2 kb, the synteny between the two genomes was visualized by Circos (v0.69-6).

### Gene Family Analysis

Coding sequences of nine species were downloaded from the NCBI and ENSEMBL databases for a gene family analysis, including the stickleback (*Gasterosteus aculeatus*), medaka (*Oryzias latipes*), tilapia (*Oreochromis niloticus*), fugu (*Takifugu rubripes*), spotted green pufferfish (*Tetraodon nigroviridis*), tongue sole (*Cynoglossus semilaevis*), zebrafish (*Danio rerio*), large yellow croaker (*Larimichthys crocea*), and spotted gar (*Lepisosteus oculatus*). BLASTP (v2.6.0+) was used to generate an alignment of all protein sequences with an *E*-value threshold of 1e − 5, and the high-quality mapped genes were analyzed by using OrthoMCL (v2.0.9) (Li et al., 2003) to define gene families. MUSCLE (v3.8.31) (Edgar, 2004) was used to align the proteins of single-copy orthologs gene families, and phase sites were extracted from each alignment and concatenated to obtain a super gene for each species. PhyML (v3.0) (Guindon et al., 2010) was used to construct phylogenetic tree. Divergence times were estimated by using MCMCtree (v4.5) in the PAML (v4.4) (Yang, 2007) package. Next, Café (v2.1) (Hahn et al., 2007) was used to define the expansion and contraction of gene families.

Gene families exhibiting expansion and contraction were mapped to KEGG pathways and GO terms for an enrichment analysis. Using the whole genome annotation results as background, hypergeometric tests were used to identify significant enrichment. Genes were extracted from the same expanded gene families and sequences were aligned using CLUSTALW (Thompson et al., 2002) of MEGA-X (v10.1) (Kumar et al., 2018). Finally, maximum likelihood tests were used to construct the phylogenetic tree.

### Detection of Positive Selection

To identify positively selected genes (PSGs) in the yellow goosefish genome, the yellow goosefish was used as the foreground branch. The coding sequences of single copy genes for the yellow goosefish and other nine species (the same species used in the gene family analysis) were extracted. PRANK (v100802) (Loytynoja, 2014) was used to generate multiple alignments of homologs genes with the parameters “ + F − codon,” and Gblocks (v0.91b) (Castresana, 2000) was used to extract conserved positions from the alignment results with the parameters “ − a = y, − c = y, w = y, − t = c, − e = gb1, − b4 = 5, − d = y.” PSGs were identified by comparing the alternative model (fix_omega = 1, omega = 1) to the null model (fix_omega = 0, omega = 1.5), and then using codeml to perform likelihood ratio tests (LRTs) at a 5% significance level implemented in PAML (v4.4). The PSGs were mapped to KEGG pathways and GO terms, and hypergeometric tests were performed using the whole gene set as background. Moreover, a Model Organisms Phenotype Enrichment Analysis (modPhEA) (Weng and Liao, 2017) was used to identify phenotypes known to be associated with the identified genes under positive selection based on a zebrafish phenotype database available at http://evol.nhri.org.tw/modPhEA/.

### RNA-Seq Analysis

RNA-seq clean data for 11 tissues were aligned to the yellow goosefish coding genes using Bowtie2 (v2.2.5) (Langmead and Salzberg, 2012). RSEM (Li and Dewey, 2011) was used to calculate gene expression levels in each sample. Differentially expressed genes (DEGs) were detected using the NOIseq (Tarazona et al., 2015) algorithm with the cutoff of foldchange of 1 and the cutoff of probability of 0.8. A gene co-expression network was constructed using the Weighted Gene Co−Expression Network Analysis (WGCNA) method (Langfelder and Horvath, 2008). The whole genome was background to perform KEGG pathway enrichment analyses of the genes of interest.

## Results and Discussion

### Genome Assembly and Annotation

A total of ∼416.79 Gb of raw data (about 554.98-fold coverage of the estimated genome size) were generated using the BGISEQ-500 platform (Mak et al., 2017), with read lengths of 100 bp for the paired-end library, 50 bp for mate-pair libraries, and 100 bp for the Hi-C library (Supplementary Table 1 and Supplementary Figure 2). After filtering raw data and estimating the genome size (Supplementary Figure 1), we assembled the genome with a total length of ∼709.23 Mb, which was close to the estimated genome size (∼750.88 Mb) after masking allelic scaffolds. The contig and scaffold N50 of the assembled genome were 164.91 kb and 32.49 Mb, respectively (Supplementary Table 2). By performing quality control of Hi-C sequencing data and implementing the 3D-DNA pipeline, we obtained 166,195,977 valid Hi-C read pairs and anchored 689.34 Mb (∼97.20%) of the assembled scaffolds to 23 clear linkage groups based on chromatin interactions (Supplementary Table 3 and Figure 1B), indicating that the karyotype number for *Lophius litulon* is 2*n* = 46, which is different from that of *Lophiomus setigerus* (2*n* = 48) (de Cascia Barreto Netto et al., 2007). To evaluate the quality of the genome assembly, we assessed the BUSCO (v3.0.2) (Simao et al., 2015) values with a complete percentage of 95.4% (Supplementary Table 4) against the actinopterygii database (odb9). We also aligned 171,254 *de novo* assembled transcripts using RNA-seq data for 11 tissues to the genome and found that 99.46% could be covered by the genome assembly (Supplementary Table 5). Together with the compact GC-depth distribution (Supplementary Figure 3), these results suggested that the high-quality genome assembly can be used as a reference genome for further analyses.

Repeat content annotation of the newly obtained genome was carried out by combining *de novo* prediction and homolog-based methods. Finally, ∼3.99 and ∼25.34% of the yellow goosefish genome sequences were recognized as tandem repeats (Supplementary Table 6) and transposable elements (TEs) (Supplementary Table 7), respectively. In addition, we integrated RNA sequencing data, homolog-based results using six species (tongue sole, zebrafish, large yellow croaker, tilapia, medaka, and fugu), and *de novo* predicted gene models, resulting in 22,382 protein-coding genes. Additionally, ∼92.80% of the genes could be assigned to known functions by mapping protein sequences to the KEGG, Swiss-Prot, TrEMBL, Interpro, and GO databases (Supplementary Table 8). Moreover, 90.6% of the complete BUSCO genes could be identified in our gene sets (Supplementary Table 4), indicating high quality of gene prediction.

### Expansion of LINEs in the Yellow Goosefish

LINEs in eukaryote genomes are important for the regulation of nearby genes and even affect phenotypes (Ong-Abdullah et al., 2015). When comparing repeat contents in the yellow goosefish genome to those of eight representative fish genomes, we found that the yellow goosefish genome contains ∼9.54% LINEs, which is similar to the content in medaka (∼10.41%) and tilapia (∼10.85%) and notably higher than those of the six other species (∼5.70% in fugu, ∼3.74% in tongue sole, ∼6.22% in three-spined stickleback, ∼4.07% in large yellow croaker, ∼4.43% in zebrafish, and ∼4.41% in spotted green pufferfish, Supplementary Table 9). In particular, LINE/L2 (51.94% of LINEs) and LINE/RTE-BovB (22.48% of LINEs) were the two most abundant subtypes among LINEs (Supplementary Figure 4). To explore the evolution of LINEs in representative fishes, phylogenetic trees of L2 and RTE-BovB were constructed. Based on these phylogenetic trees, L2 and RTE-BovB sequences can be classified into seven and three clusters, respectively (Figures 1C,D). Compared with other genomes, clusters 1 and 7 of L2, which contained elements from all the species, exhibited obvious outburst in the yellow goosefish genome. And clusters 1 and 3 of RTE-BovB also expanded significantly. Additionally, we found that RTE-BovB of cluster 3 expanded in each sub-cluster, indicating that these expansions may have occurred simultaneously during the evolutionary process, different from the pattern observed in cluster 1 exhibiting three single sub-clusters expansion in the yellow goosefish.

In summary, the yellow goosefish genome contained all L2 and RTE-BovB clusters, and these were characterized by expansions.

### Chromosome Evolution in the Yellow Goosefish

**FIGURE 2:**
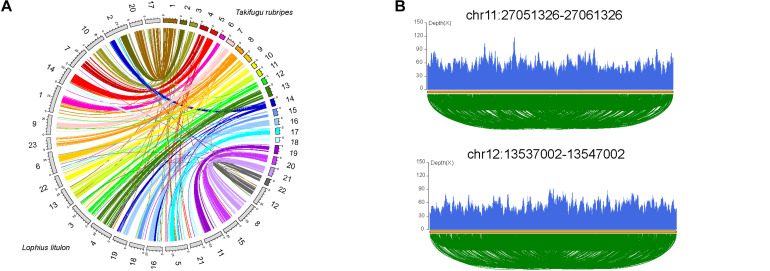
Chromosome evolution of yellow goosefish. **(A)** Synteny analysis with Takifugu rubripes. Collinear blocks between yellow goosefish and fugu. **(B)** Validation of two breakpoints by the pair end mapping of reads. More detailed information in Supplementary Material.

Chromosome evolution, including whole genome duplication, chromosome fissions/fusions/deletions/rearrangements, and random duplications of large fragments, is associated with genome size, gene family evolution, and even speciation (Li et al., 2017). We compared 23 chromosomes of yellow goosefish with 22 chromosomes of fugu (*Takifugu rubripes*, the most closely related species with a chromosome-level genome, Figure 2A, Supplementary Figure 5, and Supplementary Table 10) to investigate chromosome evolution. A total of 17 fugu chromosomes shared one-to-one synteny with distinct goosefish chromosomes, such as Fugu_20-chr15, indicating high consistency between the chromosomes of the two genomes. In addition, four fugu chromosomes could be mapped to five chromosomes of the yellow goosefish, such as Fugu_16-chr16&chr19. Thus, at least four fusion and one fission events occurred between the two species. Moreover, we identified 40 chromosomal inversion events in the yellow goosefish, which could be confirmed by sequencing data (Figure 2B and Supplementary Figure 6). We found 21 genes located in the 2 kb flanking regions of these inversion breakpoints (Supplementary Table 11) based on the alignment of the chromosomes between the yellow goosefish and the fugu. Some of these 21 genes were involved in important biological processes. For example, *Gnl2* (Lolit01435) regulates cell cycle progression and neural differentiation in the brain and retina in zebrafish (Paridaen et al., 2011).

### Topological Domains in the Genome

We identified 1535 TADs in the genome and, as expected, the domain size is largely ranged from 80 kb to 2.12 Mb (Supplementary Figure 7a), while topological boundary size expands more than 40 to 80 kb (Supplementary Figure 7b). Both topological domain and topological boundaries appear to differ from what is expected at random in terms of their gene content (Supplementary Figures 7c,d). We explored the genes near the topological boundary regions in the genome, and we found housekeeping genes were enriched in the boundaries that were reported associated with the boundary formation (Dixon et al., 2012; Eisenberg and Levanon, 2013). From the KEGG enrichment results (*p*-value < 0.05), the genes in the boundaries were enriched in pathways related to transport, catabolism, metabolism, organismal systems, and cell growth and death (Supplementary Table 12).

### Evolutionary Relationship and Gene Family Analysis

We investigated the evolutionary relationships between yellow goosefish and nine representative fish taxa (*Gasterosteus aculeatus*, *Oryzias latipes*, *Oreochromis niloticus*, *Takifugu rubripes*, *Tetraodon nigroviridis*, *Cynoglossus semilaevis*, *Danio rerio*, and *Larimichthys crocea*) using *Lepisosteus oculatus* as an outgroup based on gene families, defining 19,487 gene families in these 10 species. The yellow goosefish genome contained 6,081 single-copy orthologs and 246 unique paralogs (Supplementary Figure 8). The single copy orthologs genes were used to construct a phylogenetic tree, which was consistent with previous observations (Shao et al., 2018). The tree showed that the yellow goosefish was most closely related to Tetraodontiformes species, with a divergence time of about 95.5 million years ago (Supplementary Figure 9).

**FIGURE 3:**
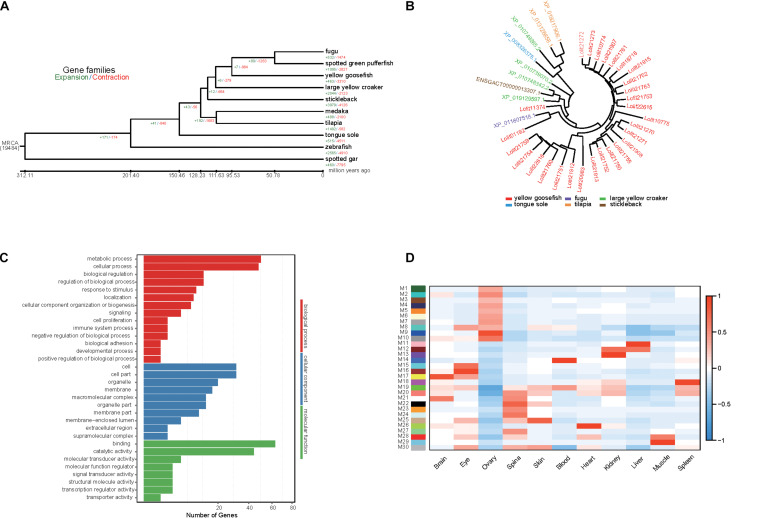
Gene family analysis, GO enrichment of PSG, and identification of gene coexpressed in yellow goosefish. **(A)** Expansion and contraction gene families in yellow goosefish and other nine species (stickleback, medaka, tilapia, fugu, spotted green pufferfish, tongue sole, zebrafish, large yellow croaker, and spotted gar). **(B)** The phylogeny of the zinc finger proteins gene family in six species (yellow goosefish, fugu, large yellow croaker, tongue sole, tilapia, and stickleback). **(C)** GO enrichment for the PSGs of yellow goosefish. Number of genes were indicated for each GO term. **(D)** WGCNA co-expression modules were constructed by comparing 11 tissues. The *x*-axis shows sampled tissues, and *y*-axis shows WGCNA modules. In total, 30 modules were identified, and the correlation value for each module ranges from –1 to 1.

Using Café, we identified 463 expanded gene families and 3,310 contracted gene families in the yellow goosefish genome (Figure 3A). We focused on 51 and 20 significantly expanded and contracted gene families (*p* < 0.01). By performing a KEGG enrichment analysis, we found that the expanded family genes were significantly enriched for pathways related to metabolism, transcription, signaling molecules and interaction, organismal systems, and human diseases (Supplementary Table 13, *p* < 0.05). In particular, homeodomain-interacting protein kinase family (HIPK), which is involved in the regulation of eye size (Inoue et al., 2010), was expanded in the yellow goosefish genome. This may explain the large eye size in the species, which may help individuals perceive and adapt to the benthic environment. In addition, we found that the KRAB domain-containing zinc finger (KRAB-ZFP)-like protein, which was reported to controls gene expression in response to environmental changes like nutrition, fasting, and hormone stimulation (Urrutia, 2003; Lupo et al., 2013), included 28 copies in yellow goosefish but fewer than four copies in other species (Figure 3B). It is possible that the expansion of the KRAB-ZFPs may help deep-sea yellow goosefish to respond to environment perturbations better. With respect to the contracted gene families, the extracellular calcium-sensing receptor family, involved in the maintenance of mineral ion homeostasis (Brown, 1999) and scale regeneration (Kakikawa et al., 2012), may be associated with the lack of scales in the yellow goosefish.

### Detection of Positively Selected Genes

Positively selected genes usually contribute to adaptive phenotypic evolution. The yellow goosefish is a bottom-dwelling fish, consistently found on the mud, sand, and gravel of the seafloor. It feeds on variety of species, requiring strong and adaptable digestive and metabolic systems (Yamada et al., 1995). Thus, we detected PSGs based on the 3,043 single-copy orthologs genes. We identified 167 PSGs containing 787 positively selected sites. We then performed a GO (Figure 3C) and KEGG enrichment analysis of these PSGs, and we found that 50 genes were enriched for metabolism-related GO terms (Supplementary Table 14) and PSGs were mainly enriched in metabolism and organismal system pathways (Supplementary Figure 10). Among these genes, *SGPL1* (sphingosine-1-phosphate lyase 1) has been reported to play a role in maintenance of lipid homeostasis in liver (Bektas et al., 2010). Two genes (*CPB1* and *PLPP2*) play important roles in protein and fat digestion and absorption (Whitcomb and Lowe, 2007; Carman and Han, 2009). *PLPP2* encodes a key enzyme that regulates the process of triacylglycerol synthesis. The liver of the yellow goosefish is largely composed of fat and is considered a delicacy; it occupies a relatively large proportion of the whole fish by weight. Positive selection on the *PLPP2* gene may reflect its important role in liver growth, but further studies are needed to confirm this hypothesis. Overall, these metabolism-related PSGs may be involved in the diverse feeding habits of the yellow goosefish, which could be an adaptation to its environment. Moreover, 30 genes were associated with specific phenotypes based on a zebrafish database by using modPhEA (Supplementary Table 15). The PSGs were significantly (*p* < 0.05) mainly associated with the phenotypes of tissue structures, compound organs, and neuron cells.

### Analysis of Transcriptomes

A total of 106.54 Gb of RNA-seq data were generated by the BGISEQ-500 platform (Supplementary Table 16). The RNAs in 11 different tissues were extracted from four yellow goosefishes. We calculated gene expression levels in each tissue and identified DEGs (Supplementary Figure 11). WGCNA (Weng and Liao, 2017) was used to identify genes expressed in concert in particular tissues based on gene expression data. A total of 30 modules (M1–M30) were identified (Figure 3D). We extracted genes from the most highly correlated module with a sample type, such as module M17 in the brain, and further identified significantly enriched KEGG pathways (*p* < 0.05) (Supplementary Figure 12). Genes in M9, M11, M22, and M26 were mainly enriched in metabolism-related pathways, including the metabolism of lipids, carbohydrates, and energy. Genes in M16 and M17 were enriched in organismal systems, mainly functional pathways related to the digestive system, nervous system, sensory system, and endocrine system. Moreover, we further investigated the genes in module M16 associated with the eye tissue. We found some genes (*PRPH2*, *RHO*, *RBP3*, and *PDE6G*) play a critical role in the visual process (Quadro et al., 1999; Toyama et al., 2008; Dvir et al., 2010; Nevo, 2013). Among these genes, *RHO* encodes rhodopsin, which is a necessary visual pigment for eyes of marine fish (Chen J. N. et al., 2018) and may be helpful in visual adaptations for yellow goosefish that live in dim light environments.

## Conclusion

In this study, we reported the first chromosome-level Lophiiformes genome, using whole genome shotgun and Hi-C sequencing data generated by the BGISEQ-500 platform. We characterized repeat elements, gene function, and chromosomal evolution. Based on the high-quality gene set, we identified the evolutionary position of the species based on a phylogenetic tree and investigated expanded gene families and PSG to find candidate loci for traits related to growth, development, and metabolism in the yellow goosefish. Our research provides a reference genome for the yellow goosefish, which is expected to be an important resource for further genetic and evolutionary studies of Lophiiformes.

## Data Availability Statement

The yellow goosefish genome assembly and annotation files have been deposited at China National GeneBank (CNGB) database with accession number CNA0007339. And all the sequencing data are available with accession number CNP0000642 at CNGB database.

## Ethics Statement

The animal study was reviewed and approved by the Institutional Review Board on Bioethics and Biosafety of BGI.

## Author Contributions

JL and CS designed and conducted the project. CLL and KL performed sample preparation and sequencing. CL and ML performed genome assembly and genome annotation. ML, YZ, and JW performed genetic analysis. ML and YZ performed RNA-seq analysis. GF, XL, HY, ML, SM, and YZ wrote the manuscript. All authors helped with the interpretation of data. All authors contributed to the article and approved the submitted version.

## Conflict of Interest

ML, YZ, CL, JW, GF, XL, HY, and JL were employed by the company BGI. The remaining authors declare that the research was conducted in the absence of any commercial or financial relationships that could be construed as a potential conflict of interest.
