# Supplementary material for: A Chromosome-Level Genome Assembly of the Anglerfish Lophius litulon
Source: Front Genet. 2020 Nov 27;11:581161. doi: 10.3389/fgene.2020.581161 (PMC7729161; doi:10.3389/fgene.2020.581161)
Supplement: Supplementary file 1 [file Data_Sheet_1.docx]

Supplementary Material

# Supplementary Figures

**Supplementary Figure 1.** K-mer frequency distribution. A total of 66.8 Gb clean sequencing data from 350bp libraries was used to conduct k-mer analysis(k=17). The genome size was estimated to be 750.88 Mb.


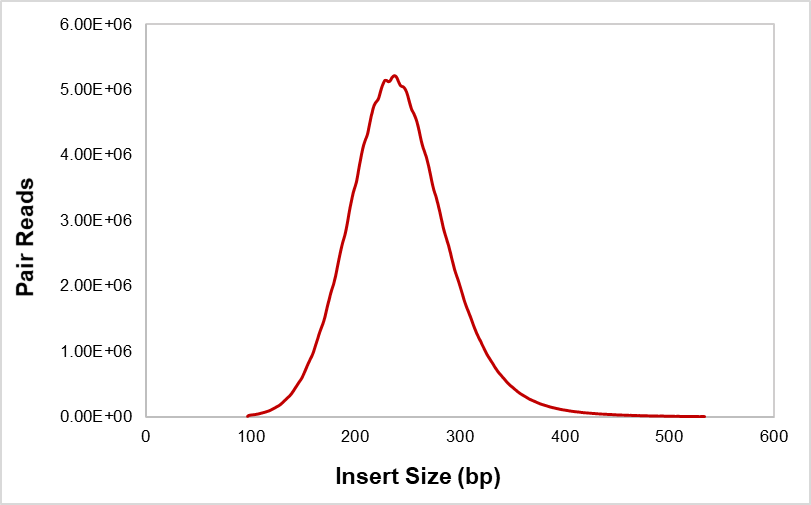


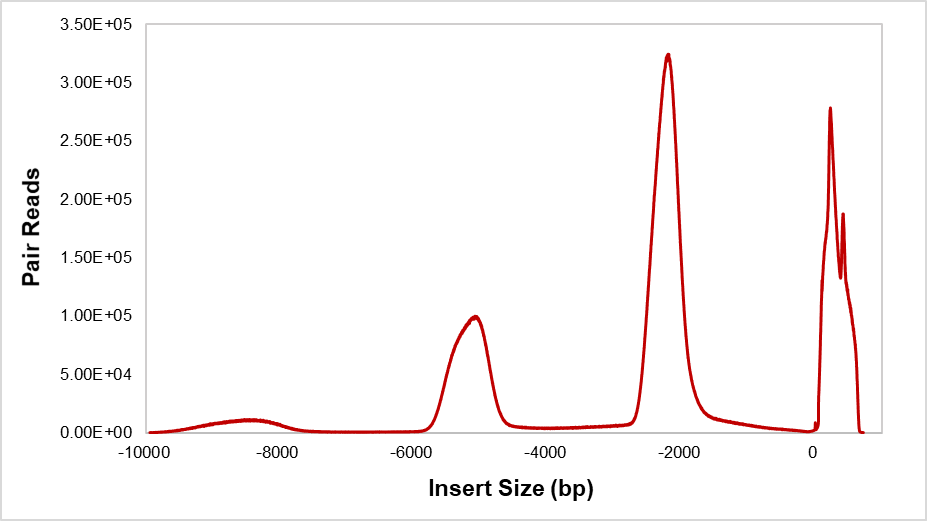


**Supplementary Figure 2.** Insert size distribution of a 350bp pair-end library and mate-pair libraries of clean reads.


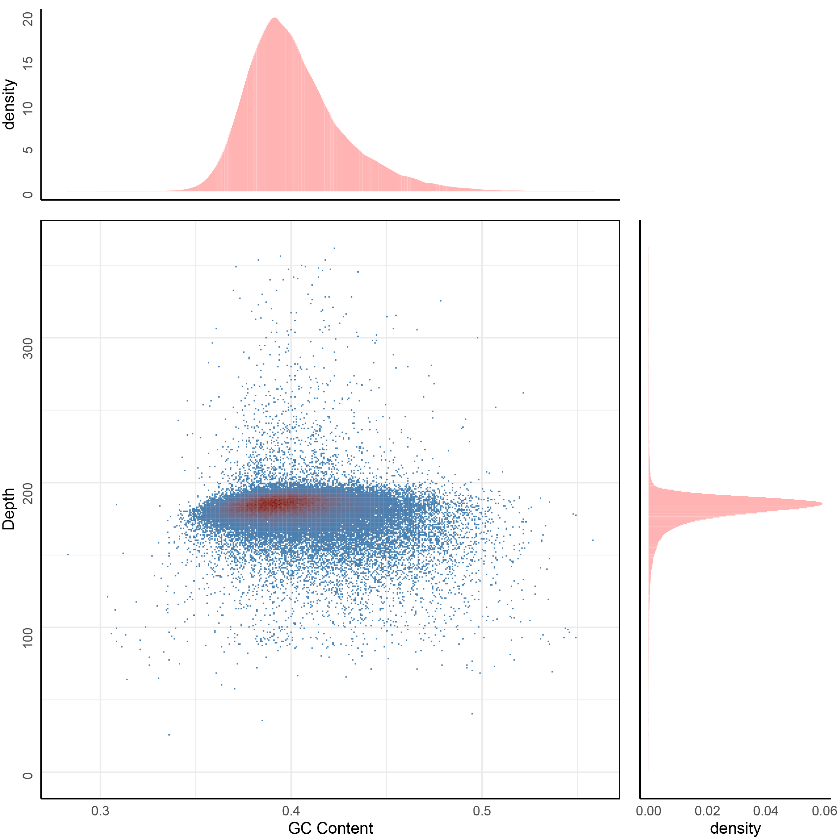


**Supplementary Figure 3.** GC-depth distribution of the yellow goosefish genome.

**Supplementary Figure 4.** The proportion of major LINE elements in the genome of nine fish species.


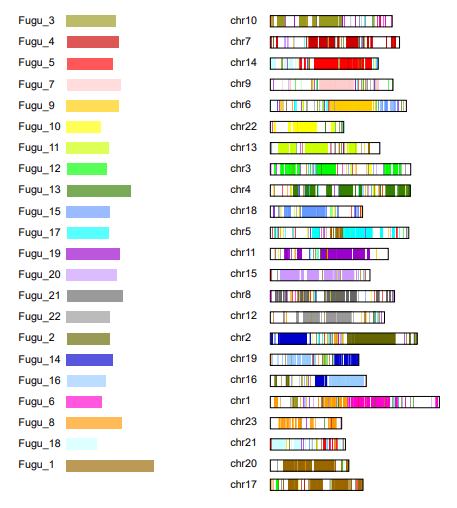


**Supplementary Figure 5.** Synteny analysis with Takifugu rubripes. Collinear blocks between yellow goosefish and fugu. Chr1-23 represents 23 chromosomes of yellow goosefish, and Fugu1-22 represents 22 chromosomes of fugu.


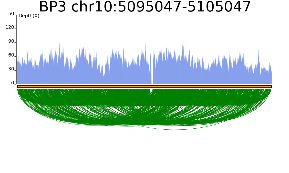

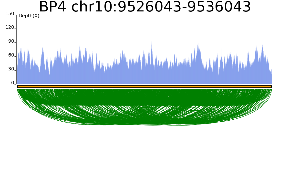

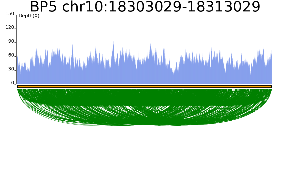

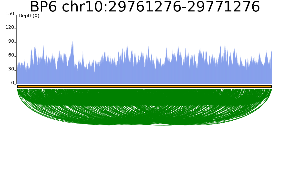

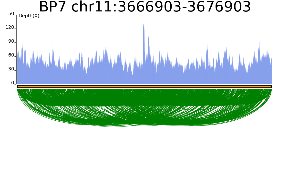

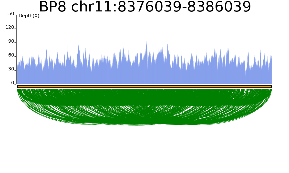

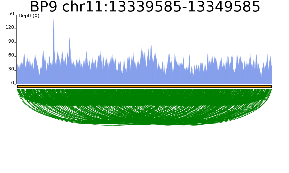

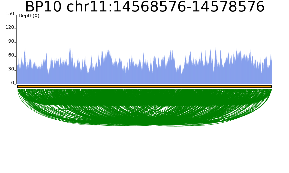

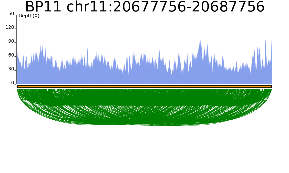

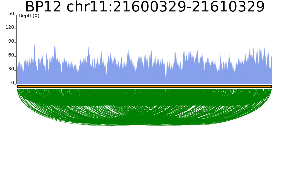

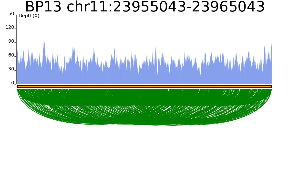

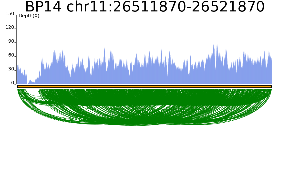

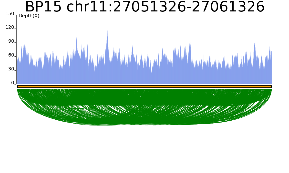

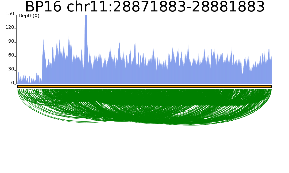

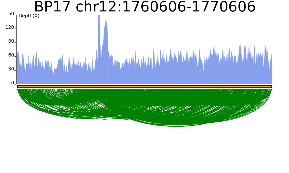

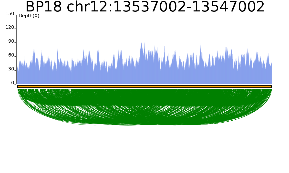

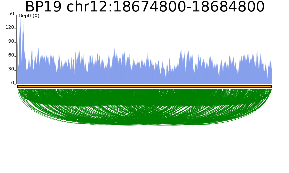

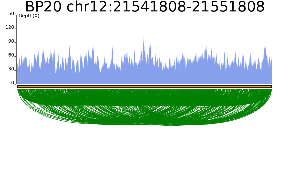

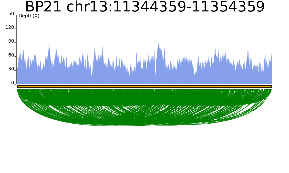

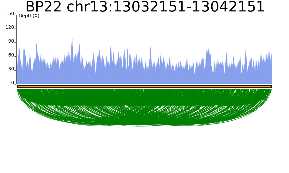

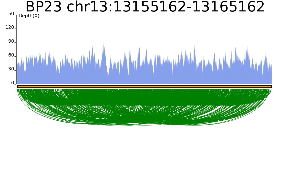

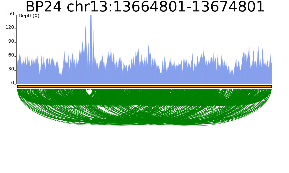

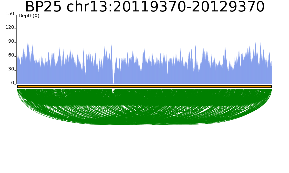

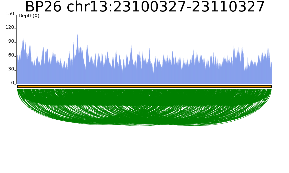

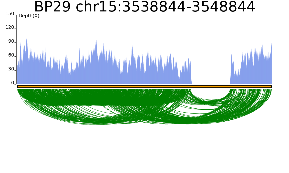

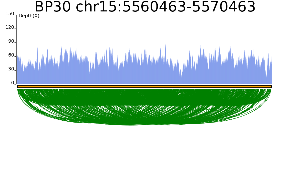

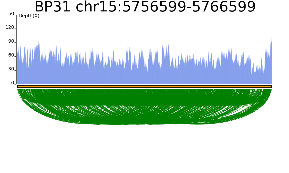

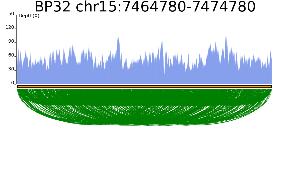

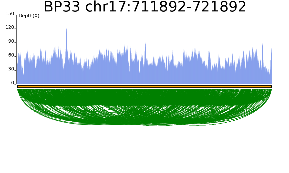

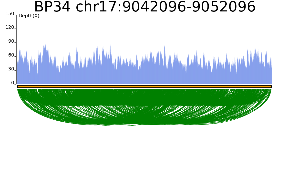

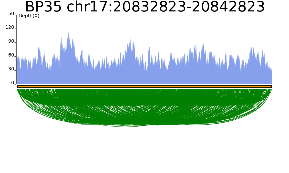

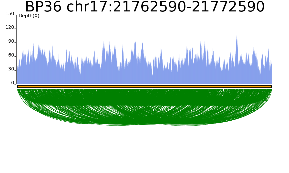

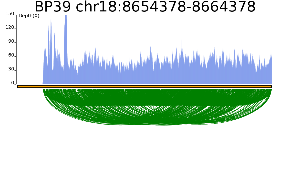

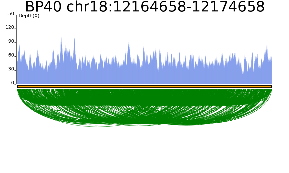

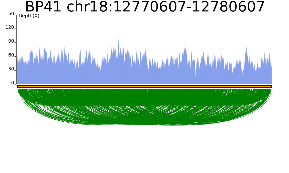

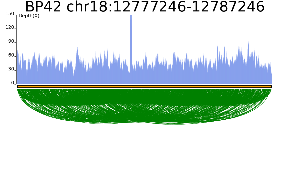

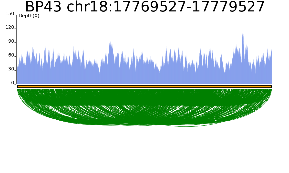

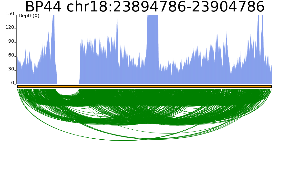

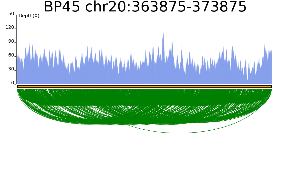

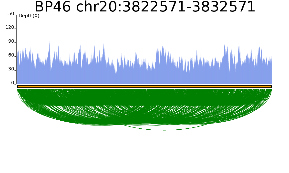

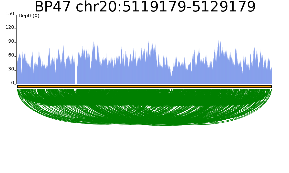

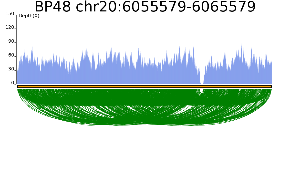

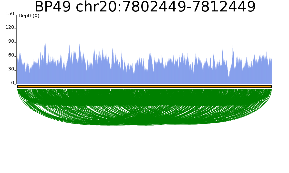

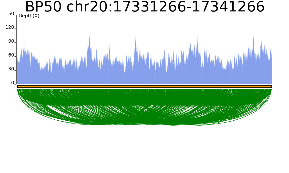

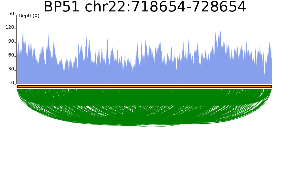

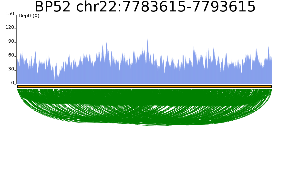

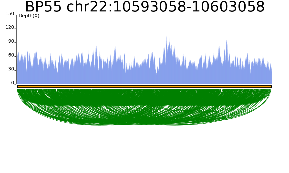

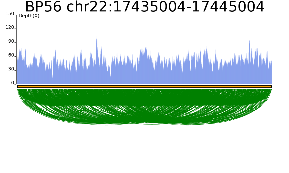

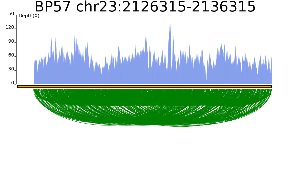

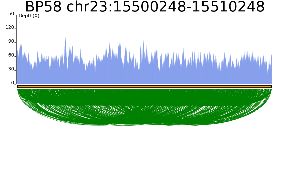

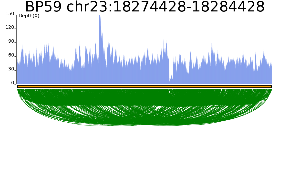

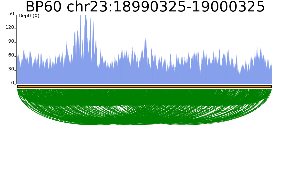

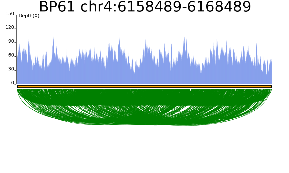

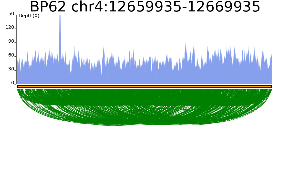

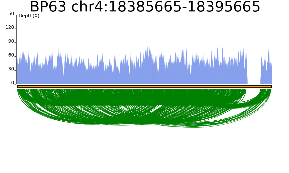

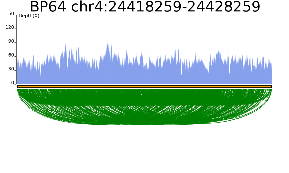

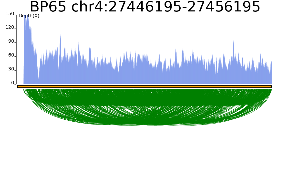

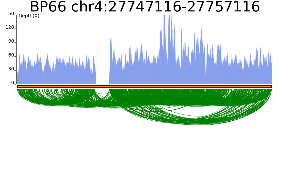

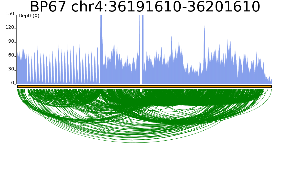

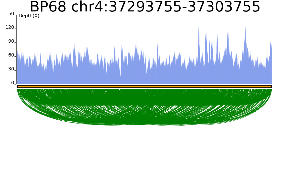

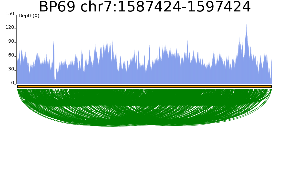

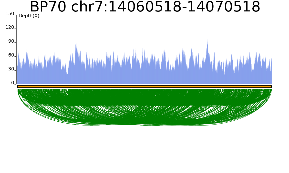

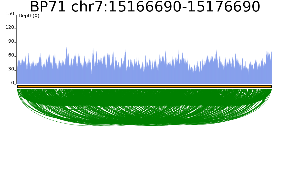

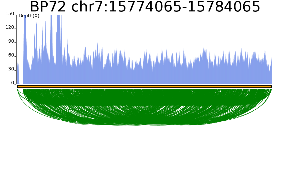

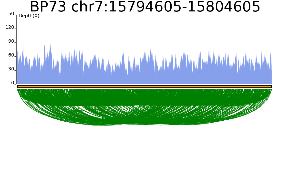

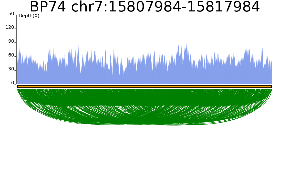

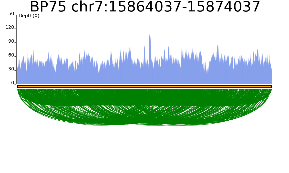

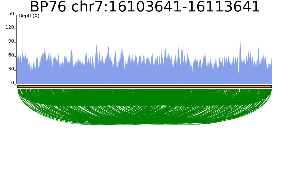


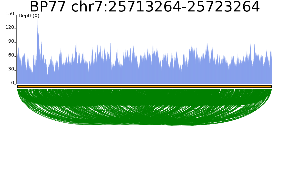

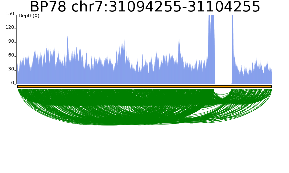

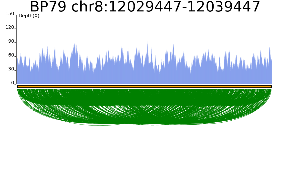

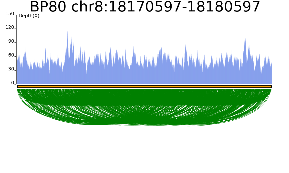


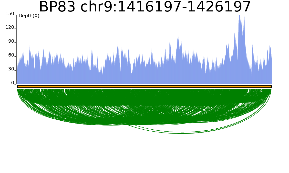

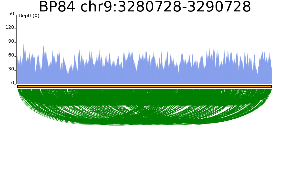

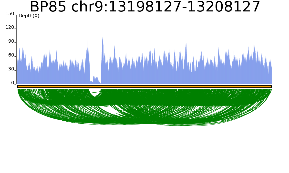

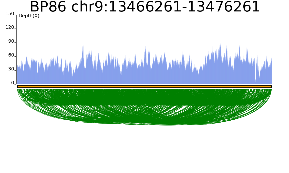


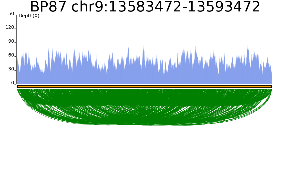

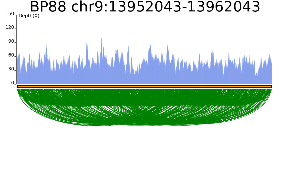

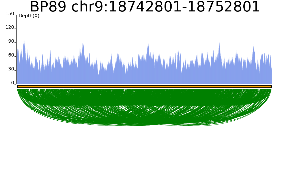

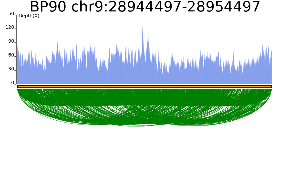


**Supplementary Figure 6.** Validation of the breakpoints by the pair end mapping of reads. We mapped reads from the libraries with insert size of 2kb and 5kb into the yellow goosefish genome to check the pair end mapping near the identified breakpoints. The vertical lines show the read depth at each position, and the curves below show the pair end information.


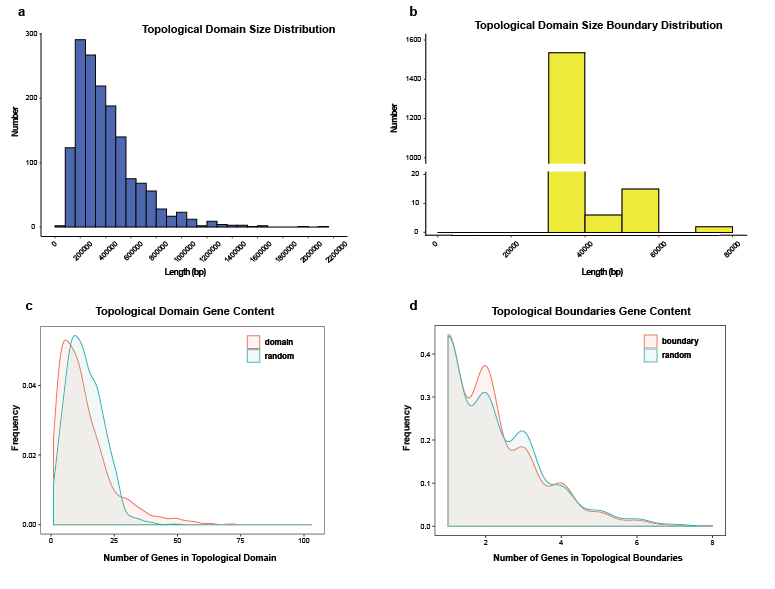


**Supplementary Figure 7.** Size distribution and gene content of topological domains, and toplogical boundaries domains. a,b) Histogram of the sizes of topological domain and toplogical boundaries domains. c,d) Distribution of the gene count for randomly chosen region of the genome with same size distribution.


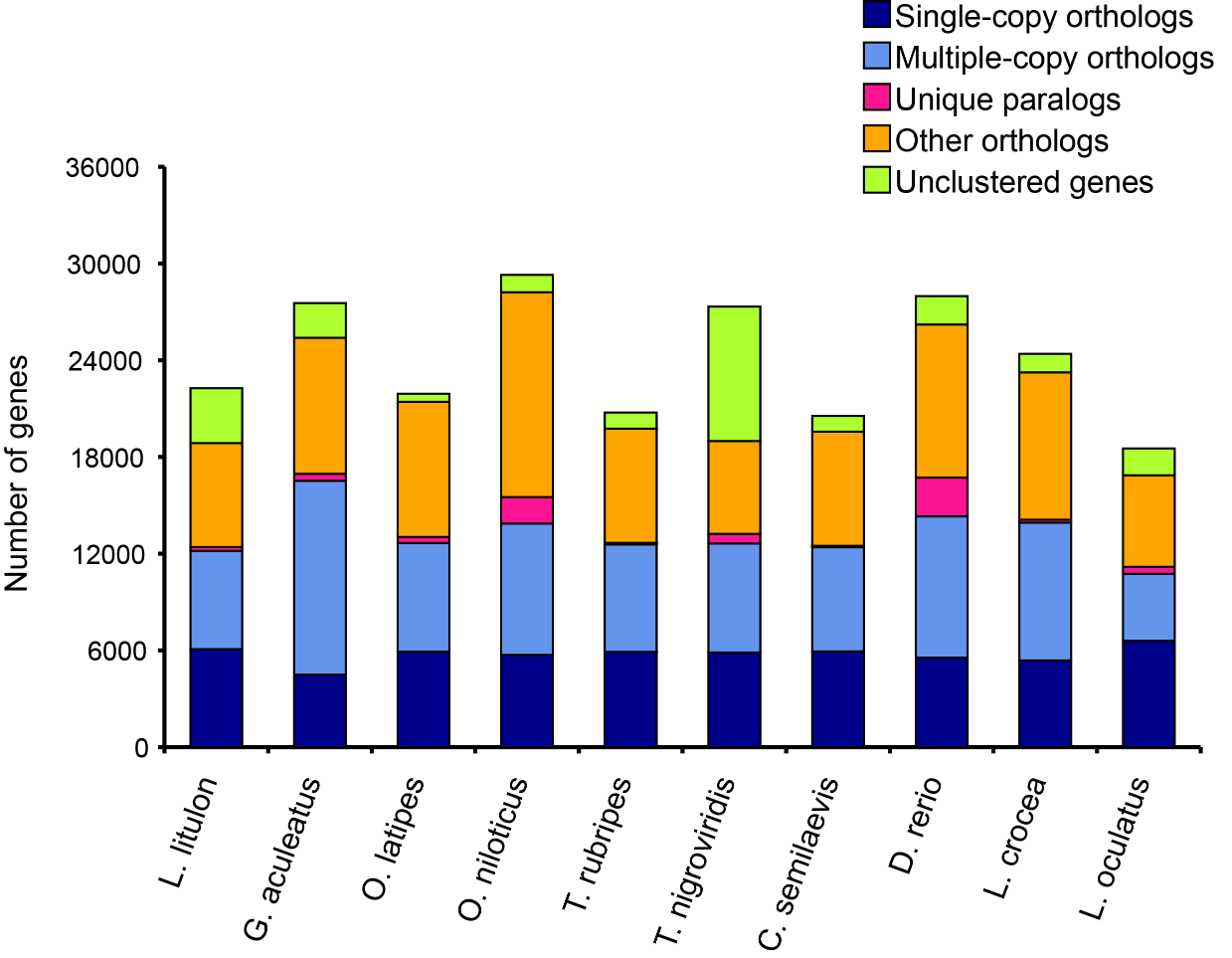


**Supplementary Figure 8.** Summary of gene family analysis. Statistics of single-copy orthologs, multiple-copy orthologs, unique paralogs, other orthologs and unclustered gene numbers in yellow goosefish and nine species (stickleback *Gasterosteus aculeatus*, medaka *Oryzias latipes*, tilapia *Oreochromis niloticus*, fugu *Takifugu rubripes*, tetradon *Tetraodon nigroviridis*, tongue sole *Cynoglossus semilaevis*, zebrafish *Danio rerio*, large yellow croaker *Larimichthys crocea* and spotted gar *Lepisosteus oculatus*).


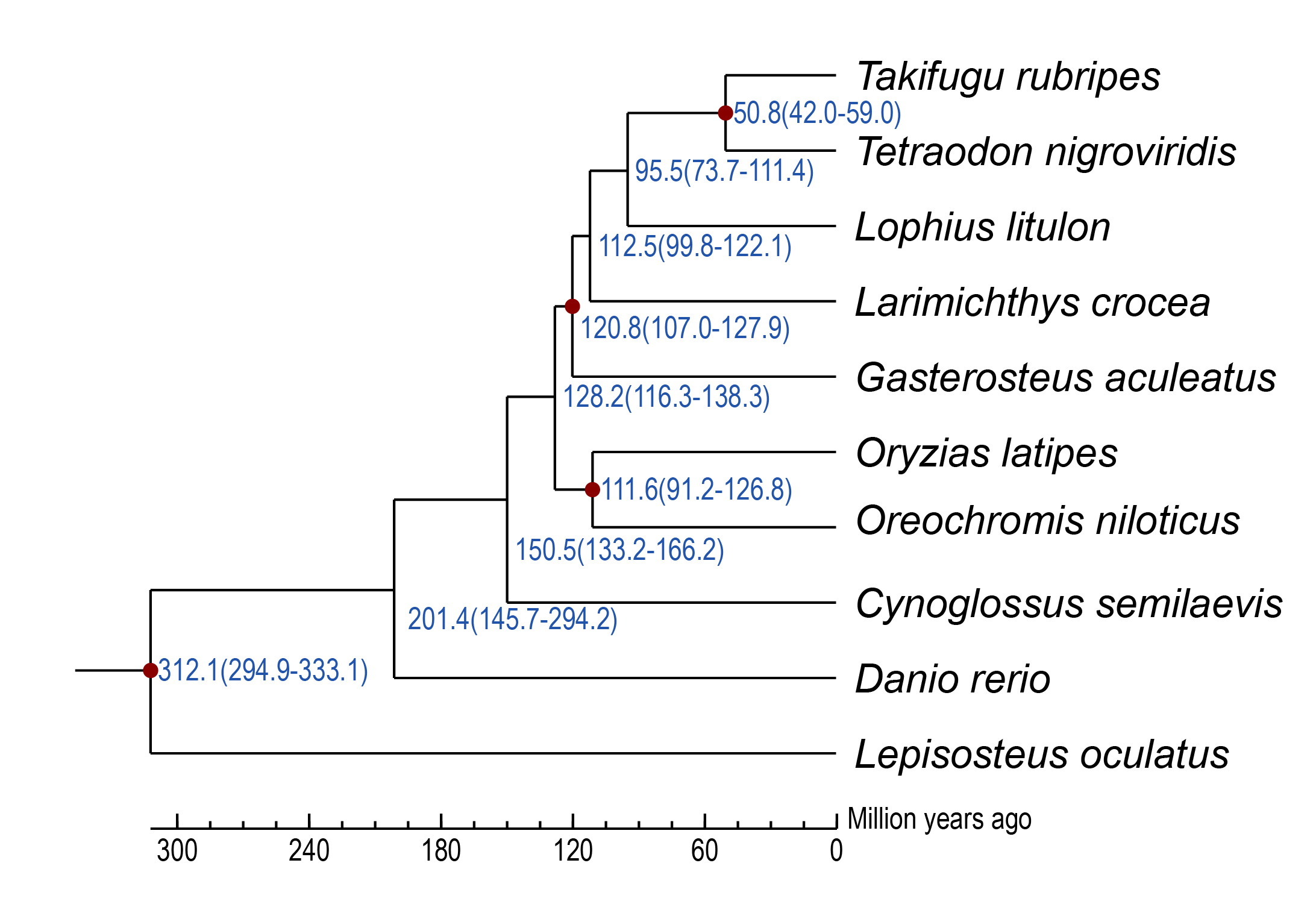


**Supplementary Figure 9.** Phylogenetic tree constructed with single copy genes. The blue numbers on the branches represent the estimated divergent times in million years.

**
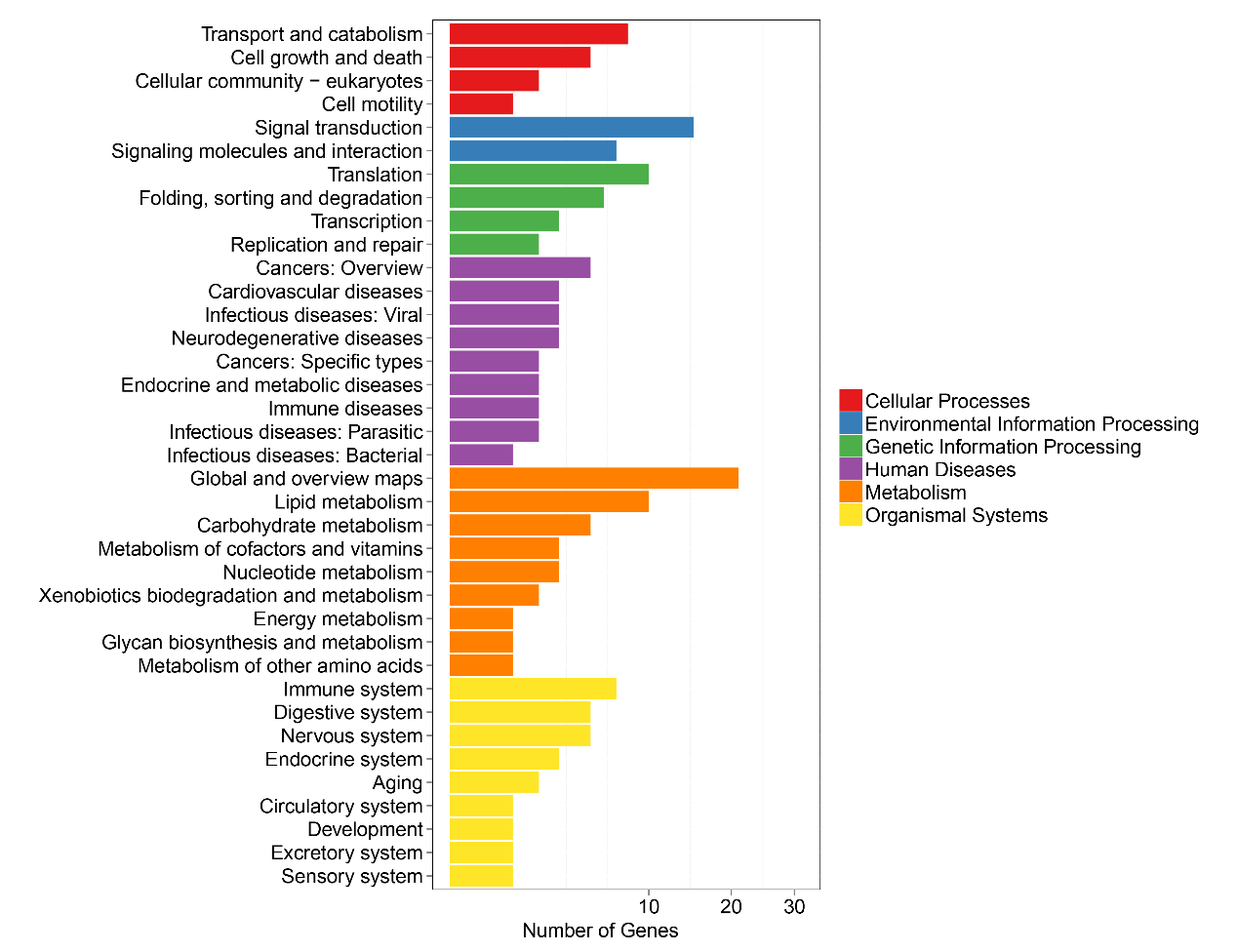
**

**Supplementary Figure 10.** KEGG pathways of genes from PSGs.

**
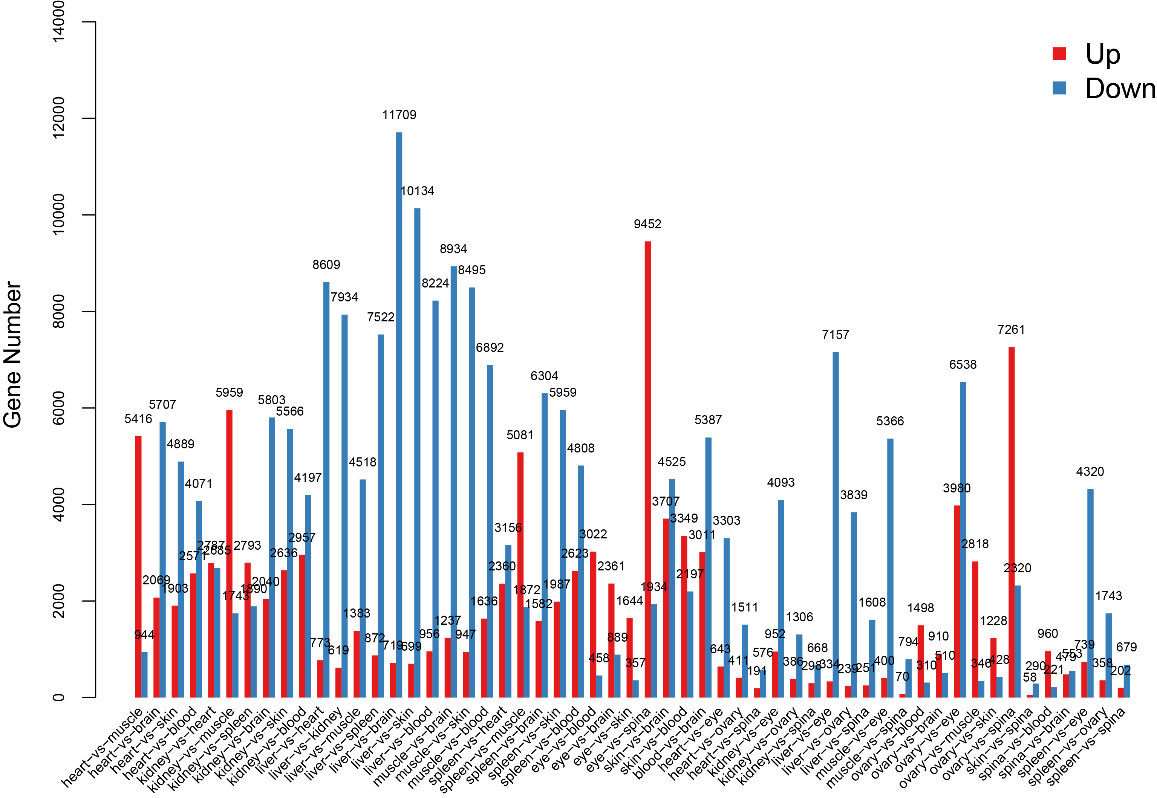
**

**Supplementary Figure 11.** Statistics of DEGs between two different tissues. The red columns represent up-regulated genes, and the blue columns represent down-regulated genes. Tissue1-vs-tissue2 means the genes have higher expression in tissue1 relative to tissue2.

**
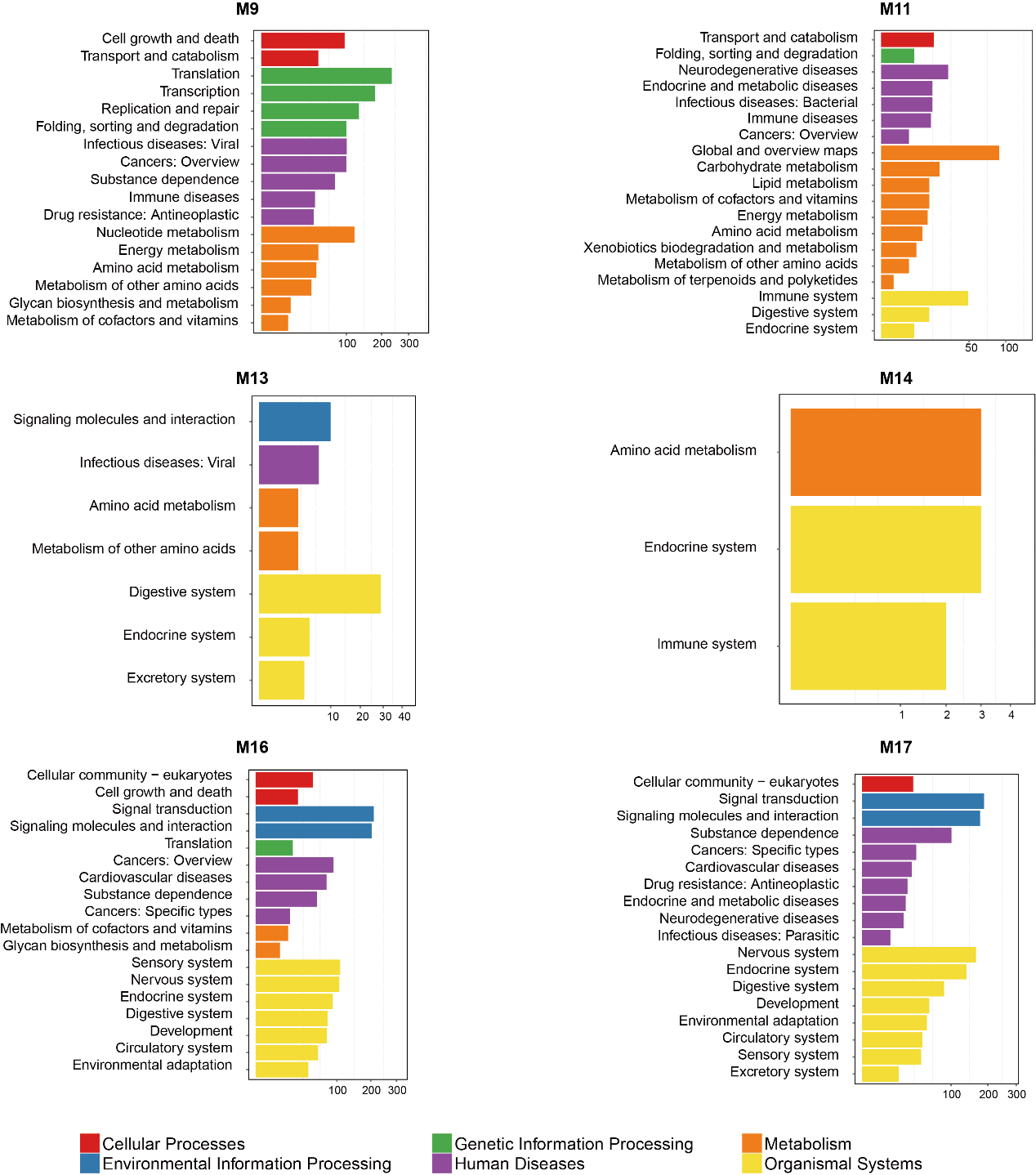
­**

**
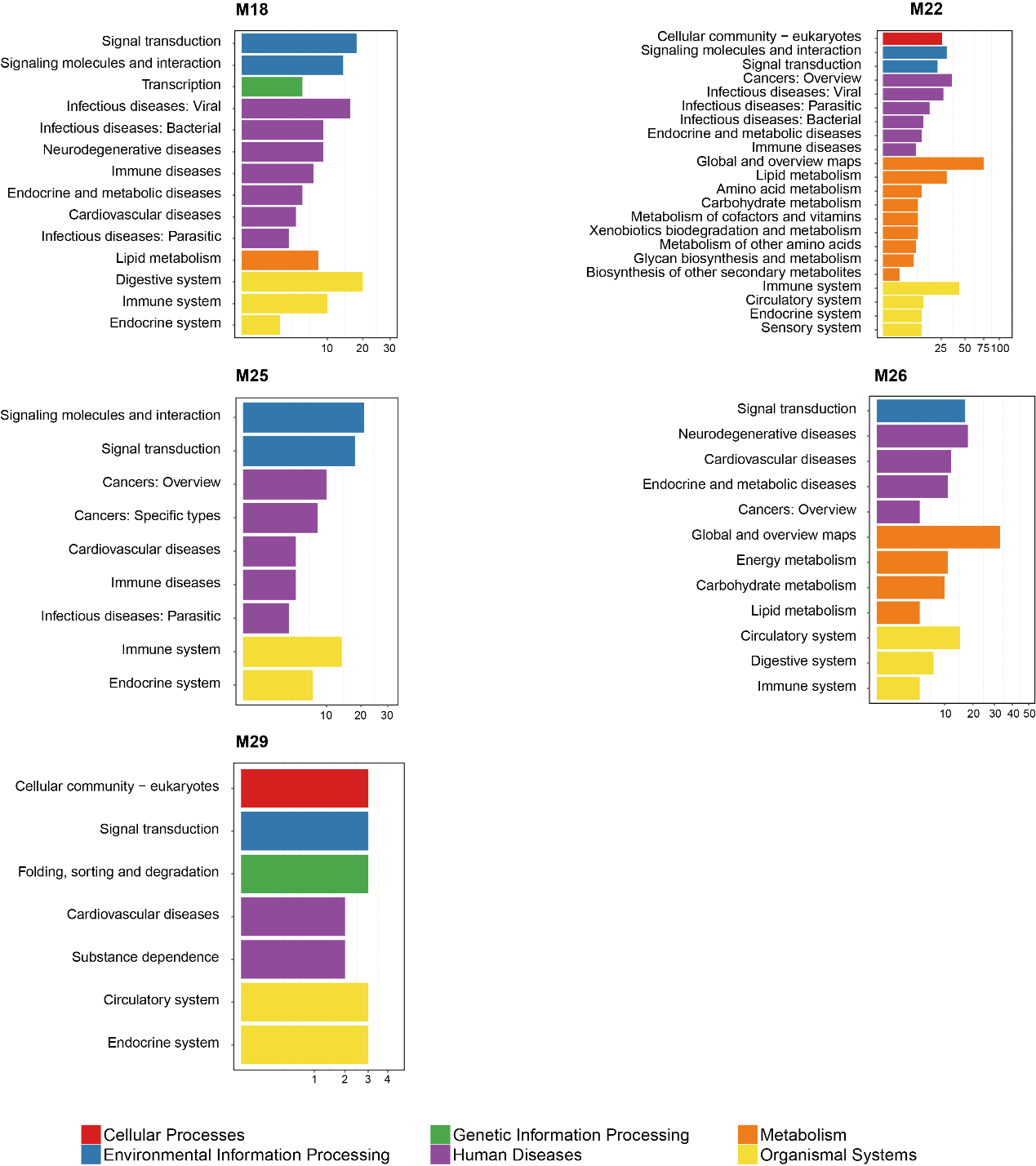
**

**Supplementary Figure 12.** KEGG pathways of genes in modules M9, M11, M13, M14, M16, M17, M18, M22, M25, M26 and M29, respectively. The X-axis indicates number of genes for each KEGG pathway.

# Supplementary Tables

**Supplementary Table 1. Summary of the sequencing data obtained for WGS and Hi-C libraries.**

| **Library** | **Insert size (bp)** | **Read length (bp)** | **Bases (Gb)** |
| --- | --- | --- | --- |
| WGS | 350 | 100 | 145.00 |
|  | 2K | 50 | 72.62 |
|  | 5K | 50 | 72.94 |
|  | 10K | 50 | 60.57 |
| Hi-C | - | 100 | 65.66 |
| Total | - | - | 416.79 |

**Supplementary Table 2. Statistics of the assembly genome information.**

| **Statistical level** | **Scaffold** | **Contig** |
| --- | --- | --- |
| Total number | 24,189 | 35,194 |
| Total length (bp) | 709,227,462 | 697,989,697 |
| Gap number (bp) | 11,237,765 | 0 |
| Average length (bp) | 29,320 | 19,833 |
| N50 Length (bp) | 32,491,500 | 164,908 |
| N90 Length (bp) | 20,022,025 | 23,490 |
| Maximum length (bp) | 45,076,811 | 1,147,210 |
| Minimum length (bp) | 200 | 63 |
| GC content (%) | 40.23 | 40.23 |

**Supplementary Table 3. Summary of the 23 chromosomes in the yellow goosefish genome.**

| **Chr** | **Length(bp)** | **Chr** | **Length(bp)** |
| --- | --- | --- | --- |
| chr1 | 45,076,811 | chr13 | 29,190,691 |
| chr2 | 39,190,480 | chr14 | 28,779,330 |
| chr3 | 37,421,109 | chr15 | 26,562,872 |
| chr4 | 37,305,391 | chr16 | 25,575,318 |
| chr5 | 36,907,915 | chr17 | 24,717,531 |
| chr6 | 36,267,886 | chr18 | 24,588,571 |
| chr7 | 34,444,954 | chr19 | 23,597,624 |
| chr8 | 33,111,908 | chr20 | 20,936,675 |
| chr9 | 32,703,222 | chr21 | 20,022,025 |
| chr10 | 32,491,500 | chr22 | 19,567,500 |
| chr11 | 31,435,303 | chr23 | 19,014,109 |
| chr12 | 30,435,481 | Total | 689,344,206 (97.20%) |

**Supplementary Table 4. BUSCO validation of the genome and geneset.**

|  | **Genome** | | **Gene** | |
| --- | --- | --- | --- | --- |
|  | **Number** | **Percentage (%)** | **Number** | **Percentage (%)** |
| Complete | 4,376 | 95.4 | 4,150 | 90.6 |
| Single-copy complete | 4,279 | 93.3 | 4,014 | 87.6 |
| Duplicated complete | 97 | 2.1 | 136 | 3.0 |
| Fragmented | 97 | 2.1 | 289 | 6.3 |
| Missing | 111 | 2.5 | 145 | 3.1 |
| Total | 4,584 | - | 4,584 | - |

**Supplementary Table 5. Coverage of *de novo* assembled transcripts using 11 tissues aligning to the genome.**

| **Dataset** | **Number** | **Total length (bp)** | **Bases covered by assembly (%)** | **Sequences covered by assembly (%)** | **with >90% sequence Number** | **in one scaffold Percent** | **with >50% sequence Number** | **in one scaffold Percent** |
| --- | --- | --- | --- | --- | --- | --- | --- | --- |
| >0bp | 171,254 | 196,114,377 | 96.14 | 99.46 | 151,089 | 88.23 | 168,930 | 98.64 |
| >200bp | 171,254 | 196,114,377 | 96.14 | 99.46 | 151,089 | 88.23 | 168,930 | 98.64 |
| >500bp | 85,262 | 170,131,553 | 95.85 | 99.75 | 71,154 | 83.45 | 83,951 | 98.46 |
| >1000bp | 53,838 | 148,070,140 | 95.61 | 99.94 | 42,799 | 79.50 | 52,911 | 98.28 |

**Supplementary Table 6. Repeat sequence statistics.**

| **Type** | **Repeat Size** | **% of genome** |
| --- | --- | --- |
| Trf | 28,287,837 | 3.99 |
| Repeatmasker | 56,146,232 | 7.92 |
| Proteinmask | 27,166,266 | 3.83 |
| De novo | 173,845,033 | 24.51 |
| Total | 197,893,094 | 27.90 |

**Supplementary Table 7. Statistics of transposable elements of the genome.**

| **Type** | **RepBase TEs** | | **TE Proteins** | | ***De novo*** | | **Combined TEs** | |
| --- | --- | --- | --- | --- | --- | --- | --- | --- |
|  | **Length (bp)** | **% in genome** | **Length (bp)** | **% in genome** | **Length (bp)** | **% in genome** | **Length (bp)** | **% in genome** |
| DNA | 29,873,814 | 4.21 | 5,398,476 | 0.76 | 68,093,431 | 9.60 | 79,456,974 | 11.20 |
| LINE | 23,133,378 | 3.26 | 20,747,214 | 2.93 | 57,841,922 | 8.16 | 67,630,350 | 9.54 |
| SINE | 3,080,854 | 0.43 | 0 | 0.00 | 2,975,117 | 0.42 | 5,178,150 | 0.73 |
| LTR | 5,858,637 | 0.83 | 1,029,660 | 0.15 | 17,799,141 | 2.51 | 22,768,603 | 3.21 |
| Other | 18,744 | 0.00 | 0 | 0.00 | 0 | 0.00 | 18,744 | 0.00 |
| Unknown | 0 | 0.00 | 0 | 0.00 | 48,357,945 | 6.82 | 48,357,945 | 6.82 |
| Total | 56,146,232 | 7.92 | 27,166,266 | 3.83 | 166,152,492 | 23.43 | 179,750,694 | 25.34 |

**Supplementary Table 8. Function annotation of the genes.**

| **Database** | **Number** | **Percent (%)** |
| --- | --- | --- |
| Total | 22,382 | 100% |
| Swiss-Prot | 19,519 | 87.21% |
| KEGG | 18,651 | 83.33% |
| TrEMBL | 20,714 | 92.55% |
| Interpro | 19,540 | 87.30% |
| GO | 14,801 | 66.13% |
| Overall | 20,771 | 92.80% |

**Supplementary Table 9. Statistics of transposable elements in nine fish genomes.**

|  | *Lophius litulon* | | *Oryzias latipes* | | *Takifugu rubripes* | | *Cynoglossus semilaevis* | | *Gasterosteus aculeatus* | |
| --- | --- | --- | --- | --- | --- | --- | --- | --- | --- | --- |
| **Type** | Length (Bp) | % in genome | Length (Bp) | % in genome | Length (Bp) | % in genome | Length (Bp) | % in genome | Length (Bp) | % in genome |
| **DNA** | 79,456,974 | 11.20 | 126,716,317 | 14.58 | 21,275,275 | 5.43 | 36,780,534 | 7.82 | 35,650,096 | 7.72 |
| **LINE** | 67,630,350 | 9.54 | 90,435,719 | 10.41 | 22,316,804 | 5.70 | 17,599,748 | 3.74 | 28,697,415 | 6.22 |
| **SINE** | 5,178,150 | 0.73 | 9,001,570 | 1.04 | 1,056,847 | 0.27 | 1,876,835 | 0.40 | 3,533,002 | 0.77 |
| **LTR** | 22,768,603 | 3.21 | 62,654,967 | 7.21 | 15,374,370 | 3.93 | 11,627,464 | 2.47 | 30,466,795 | 6.60 |
| **Other** | 18,744 | 0.00 | 2,713 | 0.00 | 6,962 | 0.00 | 5,713 | 0.00 | 1,129 | 0.00 |
| **Unknown** | 48,357,945 | 6.82 | 17,977,035 | 2.07 | 3,395,094 | 0.87 | 6,433,709 | 1.37 | 3,158,643 | 0.68 |
| **Total** | 179,750,694 | 25.34 | 253,215,905 | 29.14 | 49,743,515 | 12.71 | 59,151,167 | 12.58 | 82,399,819 | 17.85 |
|  | *Larimichthys crocea* | | *Danio rerio* | | *Oreochromis niloticus* | | *Tetraodon nigroviridis* | |  | |
| **Type** | Length (Bp) | % in genome | Length (Bp) | % in genome | Length (Bp) | % in genome | Length (Bp) | % in genome |  |  |
| **DNA** | 50,367,111 | 7.77 | 599,828,015 | 43.73 | 173,906,077 | 17.29 | 20,401,286 | 5.96 |  |  |
| **LINE** | 26,418,884 | 4.07 | 60,831,779 | 4.43 | 109,082,451 | 10.85 | 15,095,845 | 4.41 |  |  |
| **SINE** | 3,139,446 | 0.48 | 36,992,959 | 2.70 | 9,661,973 | 0.96 | 763,316 | 0.22 |  |  |
| **LTR** | 17,332,054 | 2.67 | 80,617,010 | 5.88 | 33,026,406 | 3.28 | 12,005,293 | 3.51 |  |  |
| **Other** | 8,909 | 0.00 | 3,529 | 0.00 | 97,404 | 0.01 | 9,365 | 0.00 |  |  |
| **Unknown** | 7,463,820 | 1.15 | 338,264 | 0.02 | 83,943,317 | 8.35 | 1,945,957 | 0.57 |  |  |
| **Total** | 85,823,340 | 13.24 | 818,339,975 | 59.66 | 381,667,366 | 37.95 | 38,145,150 | 11.14 |  |  |

**Supplementary Table 10. Collinearity between fugu and yellow goosefish chromosomes. Collinearity analysis was conducted using LASTZ.**

| **Chromosome**  **of fugu** | **Best blast hits in yellow goosefish chromosomes** | **Coverage** | **2^nd^ -best blast hits**  **in sperm whale**  **chromosomes** | **Coverage** |
| --- | --- | --- | --- | --- |
| Fugu_1 | chr20 | 47.34% | chr17 | 35.93% |
| Fugu_10 | chr22 | 86.00% | chr3 | 6.06% |
| Fugu_11 | chr13 | 85.45% | chr18 | 2.49% |
| Fugu_12 | chr3 | 79.46% | chr17 | 7.10% |
| Fugu_13 | chr4 | 86.97% | chr6 | 1.28% |
| Fugu_14 | chr2 | 53.69% | chr19 | 26.03% |
| Fugu_15 | chr18 | 72.17% | chr6 | 15.18% |
| Fugu_16 | chr16 | 57.56% | chr19 | 34.28% |
| Fugu_17 | chr5 | 83.27% | chr6 | 2.84% |
| Fugu_18 | chr21 | 58.94% | chr14 | 31.45% |
| Fugu_19 | chr11 | 84.27% | chr10 | 2.75% |
| Fugu_2 | chr2 | 87.49% | chr16 | 5.25% |
| Fugu_20 | chr15 | 86.38% | chr7 | 4.51% |
| Fugu_21 | chr8 | 87.41% | chr1 | 1.06% |
| Fugu_22 | chr12 | 84.94% | chr7 | 1.21% |
| Fugu_3 | chr10 | 83.70% | chr1 | 5.60% |
| Fugu_4 | chr7 | 86.07% | chr17 | 1.96% |
| Fugu_5 | chr14 | 83.01% | chr21 | 6.62% |
| Fugu_6 | chr1 | 83.04% | chr10 | 5.67% |
| Fugu_7 | chr9 | 83.82% | chr3 | 2.38% |
| Fugu_8 | chr23 | 38.81% | chr1 | 36.08% |
| Fugu_9 | chr6 | 87.05% | chr4 | 2.11% |

**Supplementary Table 11. The genes located in regions of 2 kb upstream and 2 kb downstream of the chromosome reshuffling breakpoints.**

| Gene_id | Symbol | Location | Description |
| --- | --- | --- | --- |
| Lolit19246 | dync2li1 | chr7 | Plays a role in correct intraflagellar transport and function of primary cilia. |
| Lolit19586 | vsx1 | chr7 | Regulates the expression of the cone opsin genes early in development. |
| Lolit04798 | SLC35D2 | chr8 | Served as a candidate of nucleotide sugars transporter which support energy for glycosyltransferases ([Ishida et al., 2005](#_ENREF_1)) |
| Lolit04799 | GTF2H3 | chr8 | Encodes the transcriptional factor involved in cell cycle regulation |
| Lolit01434 | DNALI1 | chr9 | Function unclear, a potential candidate for immotile cilia syndrome (ICS) in human |
| Lolit01435 | Gnl2 | chr9 | Encodes a kind of nucleolar GTP-binding proteins; may regulate cell cycle progress and neural differentiation in the brain and retina reported in zebrafish([Paridaen et al., 2011](#_ENREF_2)) |
| Lolit01787 | Snx27 | chr9 | Encodes an adaptor protein linking β2ARs to the retromer tubule and plays a role in cell’s response to the stimulus ([Temkin et al., 2011](#_ENREF_3)) |
| Lolit13035 | ntmt1 | chr10 | Encodes an N-terminal methyltransferase |
| Lolit02394 | krt8 | chr11 | Plays a role in maintaining cellular structure and cellular signal transduction |
| Lolit02463 | Pfkfb2 | chr11 | Plays a role in glycolysis |
| Lolit02950 | Arfgap1 | chr11 | Plays a role in membrane trafficking and /or vesicle transport |
| Lolit03100 | kcnk9 | chr11 | Be associated with Birk-Barel dysmorphism syndrome |
| Lolit22488 | btf3l4 | chr12 | The paralog of BTF3 which is required in transcriptional initiation |
| Lolit06936 | HKR1 | chr15 | May be relative to transcriptional regulation |
| Lolit07891 | rxrba | chr17 | A kind of retinoid-X receptor gene; mediates lipid and sugar metabolism and immune response during development of vertebrate embryos |
| Lolit08582 | dl | chr17 | Serves as a dopamine receptor |
| Lolit09114 | mpp1 | chr18 | May interact on regulation of neutrophil polarization |
| Lolit09136 | ABCG4 | chr18 | A member of the ATP-binding cassette (ABC) transporter superfamily; plays a role in cellular cholesterol homeostasis |
| Lolit09562 | H3 | chr18 | Histone H3 |
| Lolit12207 | POFUT2 | chr20 | Catalyzes the fucose transfer |
| Lolit14101 | RBM4B | chr23 | RNA binding motif protein |

**Supplementary Table 12. KEGG enrichment for genes in TAD boundary regions.**

| **KEGG Pathway ID** | **KEGG Pathway name** | **P value** |
| --- | --- | --- |
| ko04142 | Lysosome | 0.0015 |
| ko05134 | Legionellosis | 0.0031 |
| ko00130 | Ubiquinone and other terpenoid-quinone biosynthesis | 0.0099 |
| ko04672 | Intestinal immune network for IgA production | 0.0115 |
| ko05340 | Primary immunodeficiency | 0.0170 |
| ko04210 | Apoptosis | 0.0195 |
| ko04744 | Phototransduction | 0.0220 |
| ko05016 | Huntington's disease | 0.0228 |
| ko04620 | Toll-like receptor signaling pathway | 0.0233 |
| ko03320 | PPAR signaling pathway | 0.0298 |
| ko05320 | Autoimmune thyroid disease | 0.0328 |
| ko03022 | Basal transcription factors | 0.0366 |
| ko05330 | Allograft rejection | 0.0374 |
| ko05322 | Systemic lupus erythematosus | 0.0405 |
| ko04977 | Vitamin digestion and absorption | 0.0435 |
| ko04726 | Serotonergic synapse | 0.0448 |

**Supplementary Table 13. KEGG enrichment for genes of expanded gene families of yellow goosefish.**

| **KEGG Pathway ID** | **KEGG Pathway name** | **P value** |
| --- | --- | --- |
| ko05033 | Nicotine addiction | 8.29E-10 |
| ko03020 | RNA polymerase | 9.68E-10 |
| ko04974 | Protein digestion and absorption | 1.38E-08 |
| ko04514 | Cell adhesion molecules (CAMs) | 3.40E-07 |
| ko04725 | Cholinergic synapse | 1.12E-06 |
| ko00240 | Pyrimidine metabolism | 4.99E-06 |
| ko05169 | Epstein-Barr virus infection | 5.67E-05 |
| ko00230 | Purine metabolism | 7.38E-05 |
| ko05168 | Herpes simplex infection | 0.0001 |
| ko05016 | Huntington's disease | 0.0008 |
| ko05150 | Staphylococcus aureus infection | 0.0008 |
| ko05310 | Asthma | 0.0014 |
| ko05146 | Amoebiasis | 0.0022 |
| ko00603 | Glycosphingolipid biosynthesis - globo and isoglobo series | 0.0022 |
| ko04722 | Neurotrophin signaling pathway | 0.0026 |
| ko04080 | Neuroactive ligand-receptor interaction | 0.0028 |
| ko05332 | Graft-versus-host disease | 0.0045 |
| ko05330 | Allograft rejection | 0.0087 |
| ko04672 | Intestinal immune network for IgA production | 0.0094 |
| ko04933 | AGE-RAGE signaling pathway in diabetic complications | 0.0095 |
| ko05416 | Viral myocarditis | 0.0119 |
| ko00601 | Glycosphingolipid biosynthesis - lacto and neolacto series | 0.0154 |
| ko05221 | Acute myeloid leukemia | 0.0174 |
| ko05320 | Autoimmune thyroid disease | 0.0175 |
| ko05110 | Vibrio cholerae infection | 0.0219 |
| ko04940 | Type I diabetes mellitus | 0.0220 |
| ko00515 | Mannose type O-glycan biosynthesis | 0.0233 |
| ko05130 | Pathogenic Escherichia coli infection | 0.0323 |
| ko05144 | Malaria | 0.0408 |
| ko02010 | ABC transporters | 0.0442 |

**Supplementary Table 14. GO enrichment for the PSGs of** **yellow goosefish.**

| GO Terms | Description | Gene number | P-value |
| --- | --- | --- | --- |
| GO:1901360 | organic cyclic compound metabolic process | 22 | 5.53E-05 |
| GO:0006725 | cellular aromatic compound metabolic process | 21 | 0.00013 |
| GO:0046483 | heterocycle metabolic process | 21 | 0.00013 |
| GO:0034641 | cellular nitrogen compound metabolic process | 23 | 0.00016 |
| GO:0016070 | RNA metabolic process | 14 | 0.00018 |
| GO:0090304 | nucleic acid metabolic process | 17 | 0.00021 |
| GO:0006807 | nitrogen compound metabolic process | 24 | 0.00034 |
| GO:0006139 | nucleobase-containing compound metabolic process | 19 | 0.00092 |
| GO:0071704 | organic substance metabolic process | 40 | 0.01864 |
| GO:0034660 | ncRNA metabolic process | 6 | 0.02058 |
| GO:0008152 | metabolic process | 44 | 0.05364 |
| GO:0044238 | primary metabolic process | 37 | 0.11606 |
| GO:0043170 | macromolecule metabolic process | 31 | 0.16425 |
| GO:0044260 | cellular macromolecule metabolic process | 26 | 0.33252 |
| GO:0044237 | cellular metabolic process | 31 | 0.4009 |
| GO:0072527 | pyrimidine-containing compound metabolic process | 2 | 1 |
| GO:0016071 | mRNA metabolic process | 3 | 1 |
| GO:0006399 | tRNA metabolic process | 3 | 1 |
| GO:1901564 | organonitrogen compound metabolic process | 9 | 1 |
| GO:0006022 | aminoglycan metabolic process | 2 | 1 |
| GO:0051186 | cofactor metabolic process | 3 | 1 |
| GO:0045934 | negative regulation of nucleobase-containing compound metabolic process | 2 | 1 |
| GO:0051172 | negative regulation of nitrogen compound metabolic process | 2 | 1 |
| GO:0031324 | negative regulation of cellular metabolic process | 2 | 1 |
| GO:0009892 | negative regulation of metabolic process | 2 | 1 |
| GO:0006732 | coenzyme metabolic process | 2 | 1 |
| GO:0005975 | carbohydrate metabolic process | 4 | 1 |
| GO:0019693 | ribose phosphate metabolic process | 2 | 1 |
| GO:0006259 | DNA metabolic process | 3 | 1 |
| GO:1901135 | carbohydrate derivative metabolic process | 4 | 1 |
| GO:0006518 | peptide metabolic process | 3 | 1 |
| GO:0043603 | cellular amide metabolic process | 3 | 1 |
| GO:0009117 | nucleotide metabolic process | 2 | 1 |
| GO:0006753 | nucleoside phosphate metabolic process | 2 | 1 |
| GO:0019637 | organophosphate metabolic process | 3 | 1 |
| GO:0055086 | nucleobase-containing small molecule metabolic process | 2 | 1 |
| GO:0019538 | protein metabolic process | 15 | 1 |
| GO:0044281 | small molecule metabolic process | 4 | 1 |
| GO:0019752 | carboxylic acid metabolic process | 2 | 1 |
| GO:0044267 | cellular protein metabolic process | 11 | 1 |
| GO:0006082 | organic acid metabolic process | 2 | 1 |
| GO:0043436 | oxoacid metabolic process | 2 | 1 |
| GO:0019219 | regulation of nucleobase-containing compound metabolic process | 7 | 1 |
| GO:0044710 | single-organism metabolic process | 9 | 1 |
| GO:0051171 | regulation of nitrogen compound metabolic process | 7 | 1 |
| GO:0080090 | regulation of primary metabolic process | 7 | 1 |
| GO:0031323 | regulation of cellular metabolic process | 7 | 1 |
| GO:0051252 | regulation of RNA metabolic process | 6 | 1 |
| GO:0019222 | regulation of metabolic process | 7 | 1 |
| GO:0006796 | phosphate-containing compound metabolic process | 6 | 1 |
| GO:0006793 | phosphorus metabolic process | 6 | 1 |
| GO:0060255 | regulation of macromolecule metabolic process | 6 | 1 |

**Supplementary Table 15. Specific phenotypes identified associated with positive selected genes based on zebrafish database by using modPhEA.**

| **Phenotype Name** | **Phenotype ID** | **Fisher's Exact Test P-value** |
| --- | --- | --- |
| bile canaliculus | ZFA:0005163 | 0.045 |
| whole organism | ZFA:0001094 | 0.026 |
| organism subdivision | ZFA:0001308 | 0.026 |
| trunk | ZFA:0001115 | 0.049 |
| dorso-rostral cluster | ZFA:0007001 | 0.03 |
| compact layer of ventricle | ZFA:0005062 | 0.038 |
| pancreas | ZFA:0000140 | 0.034 |
| peripheral olfactory organ | ZFA:0000047 | 0.012 |
| olfactory bulb protoglomerulus | ZFA:0005668 | 0.008 |
| lateral protoglomerulus | ZFA:0005669 | 0.008 |
| lateral protoglomerulus 1 | ZFA:0005717 | 0.008 |
| lateral protoglomerulus 2 | ZFA:0005718 | 0.008 |
| serous membrane | ZFA:0005425 | 0.036 |
| pericardium | ZFA:0000054 | 0.035 |
| white matter | ZFA:0001682 | 0.041 |
| telencephalic white matter | ZFA:0000597 | 0.028 |
| anterior commissure | ZFA:0001108 | 0.026 |
| postoptic commissure | ZFA:0000059 | 0.028 |
| primary head sinus | ZFA:0005026 | 0.015 |
| gut | ZFA:0000112 | 0.02 |
| inner ear | ZFA:0000217 | 0.036 |
| terminal Schwann cell | ZFA:0009298 | 0.008 |
| pigment cell | ZFA:0009090 | 0.023 |
| melanocyte | ZFA:0009091 | 0.034 |
| efferent neuron | ZFA:0009239 | 0.036 |
| motor neuron | ZFA:0009052 | 0.027 |
| VaP motor neuron | ZFA:0005181 | 0.038 |
| MiD2cm | ZFA:0005185 | 0.008 |
| MiD3cl | ZFA:0005195 | 0.008 |
| MiD3cm | ZFA:0005196 | 0.023 |
| RoL2 | ZFA:0005198 | 0.023 |
| CoPA | ZFA:0005175 | 0.03 |

**Supplementary Table 16. Summary of the RNA sequencing data from 11 tissues of three yellow goosefishes.**

| **Sample** | **Tissue** | | **Total Raw Bases (Gb)** | **Total Clean Bases (Gb)** |
| --- | --- | --- | --- | --- |
| f1_Br_1A | | Brain | 6.52 | 6.21 |
| f1_Ey_1A | | Eye | 5.04 | 4.68 |
| f1_Ov_1A | | Ovary | 5.14 | 4.75 |
| f1_Ov_1B | | Ovary | 5.87 | 5.53 |
| f1_Sd_1A | | Spina | 5.96 | 5.71 |
| f1_Sd_2A | | Spina | 4.70 | 4.44 |
| f1_Sd_3A | | Spina | 6.10 | 5.71 |
| f1_Sk_1A | | Skin | 6.31 | 5.95 |
| f2_Ey_1A | | Eye | 5.37 | 5.01 |
| f2_Ov_1A | | Ovary | 5.65 | 5.25 |
| f2_Ov_1B | | Ovary | 6.61 | 6.19 |
| f3_Bl_1A | | Blood | 5.37 | 5.03 |
| f3_Ov_1A | | Ovary | 5.09 | 4.88 |
| f3_Ov_1B | | Ovary | 6.22 | 5.97 |
| Ankang_heart_A | | Heart | 7.58 | 7.05 |
| Ankang_kidney_A | | Kidney | 6.60 | 6.19 |
| Ankang_liver_A | | Liver | 4.61 | 4.34 |
| Ankang_muscle_B | | Muscle | 4.78 | 4.46 |
| Ankang_ovary_A | | Ovary | 4.39 | 4.06 |
| Ankang_spleen_A | | Spleen | 5.50 | 5.13 |
| Total | | - | 113.43 | 106.54 |

**Reference**

Ishida, N., Kuba, T., Aoki, K., Miyatake, S., Kawakita, M., and Sanai, Y. (2005). Identification and characterization of human Golgi nucleotide sugar transporter SLC35D2, a novel member of the SLC35 nucleotide sugar transporter family. *Genomics* 85**,** 106-116.

Paridaen, J.T., Janson, E., Utami, K.H., Pereboom, T.C., Essers, P.B., Van Rooijen, C., Zivkovic, D., and Macinnes, A.W. (2011). The nucleolar GTP-binding proteins Gnl2 and nucleostemin are required for retinal neurogenesis in developing zebrafish. *Dev Biol* 355**,** 286-301.

Temkin, P., Lauffer, B., Jager, S., Cimermancic, P., Krogan, N.J., and Von Zastrow, M. (2011). SNX27 mediates retromer tubule entry and endosome-to-plasma membrane trafficking of signalling receptors. *Nat Cell Biol* 13**,** 715-721.
